# Metal-Free Curing of 3D Printable Silicone Elastomers via Thermally Triggered 2-Oxazoline Cross-Linkers

**DOI:** 10.1021/acs.macromol.4c03121

**Published:** 2025-02-18

**Authors:** Paul Strasser, Christina Walliser, Edip Ajvazi, Felix Bauer, Oliver Brüggemann, Sebastian Lämmermann, Zoltan Major, Alžbeta Minarčíková, Monika Majerčíková, Matej Mičušík, Angela Kleinová, Zuzana Kroneková, Juraj Kronek, Ian Teasdale

**Affiliations:** † Institute of Polymer Chemistry, Johannes Kepler University Linz, Altenberger Straße 69, 4040 Linz, Austria; ‡ Institute of Polymer Product Engineering, Johannes Kepler University Linz, Altenberger Straße 69, 4040 Linz, Austria; § Department for Biomaterials Research, Polymer Institute, Slovak Academy of Sciences, Dúbravská cesta 9, 845 41 Bratislava, Slovakia

## Abstract

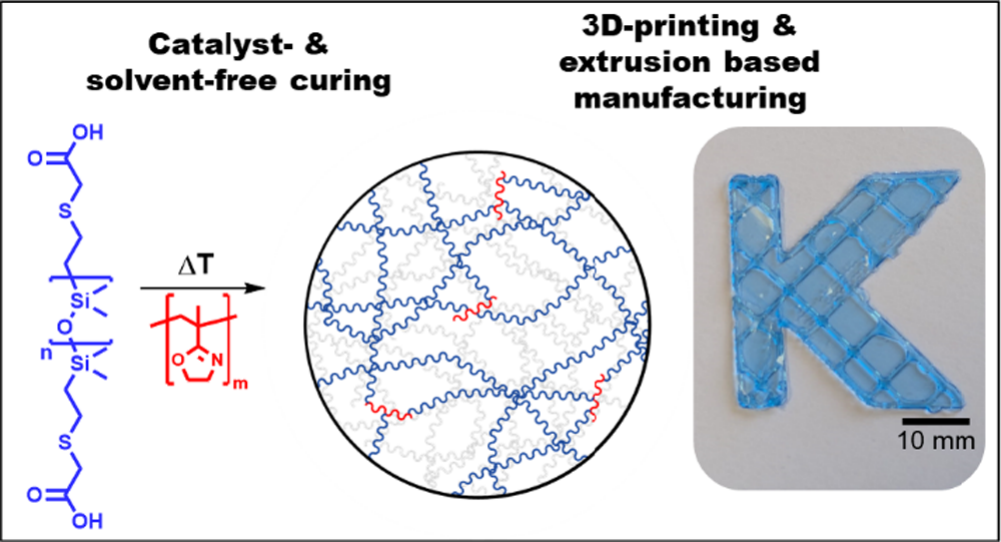

Silicone elastomers are commonly cured by hydrosilylation or condensation reactions, both of which require metal-based catalysts. Due to environmental, toxicological, and cost concerns, there is considerable interest in developing metal-free alternatives. Herein, we present a novel solution via an atom-efficient, catalyst-free ring-opening of poly(2-isopropenyl-2-oxazoline) (PiPOx) as a curing agent. PiPOx are macromolecules bearing pendant reactive 2-oxazoline groups capable of undergoing a ring-opening reaction in the presence of carboxylic acids to give covalent amide-ester bonds. Polydimethylsiloxane (PDMS) chains with COOH moieties at the chain ends could be effectively cured with PiPOx as a latent curing agent. The reaction does not proceed at room temperature but cures in less than 5 min at elevated temperatures (>80 °C) in the absence of catalysts or solvents and without the evolution of volatiles. The PDMS diacids are easily accessible via a simple thiolene addition to divinyl siloxanes in a single step, thus extending the utility of this approach to all divinyl siloxanes, which are widely commercially available in a broad range of chain lengths. The cured elastomers contain up to 98 wt % of the PDMS constituent (including the end-groups), hence mirroring the chemical structure of traditional metal-cured commercial PDMS elastomers. Solvent-free, thermally triggered curing in a practical temperature range facilitates processing using standard extrusion-based processing and additive manufacturing techniques.

## Introduction

Polysiloxanes and, in particular, polydimethylsiloxane (PDMS) are of great importance in various scientific fields with a broad spectrum of applications. They are one of the most prominent inorganic polymers, offering unique physical and mechanical properties in combination with chemical and thermal stabilities and biocompatibility.^1,2^ Polysiloxanes constitute an indispensable material not only in adhesives, sealants, automotive or electronic sectors, but also in biomedical devices, pharmacy, or personal care products.^3−7^ Especially, cured elastomers, with a market share of 41% of the global silicone market (21.3 billion dollars market size) in 2023,^8^ are of great industrial interest.

Conventionally, PDMS is either cross-linked via a condensation or addition/hydrosilylation reaction, necessitating metal catalysts such as tin or platinum, respectively.^1,9^ Furthermore, while condensation curing allows for a simple one-component system, long curing times and elimination side products limit its applicability. On the other hand, hydrosilylation reactions decrease curing times but require more complex and expensive two-component formulations. Nevertheless, the major drawbacks of both approaches persist with the metal catalysts. Although catalyst contents have been continually decreased down to ppm concentrations, an overall consumption of ∼5 tonnes of platinum per year can be attributed to silicone elastomers.^10,11^ Additionally, the utilized catalyst will remain in the cured polymer network, which has raised concerns in some biomedical and optical applications.^9,12^ Finally, the sensitive metal catalysts also limit the chemical versatility incorporated in PDMS formulations by poisoning the catalysts through, for example, nitrogen, phosphorus, or sulfur-containing functional groups.^13,14^

Driven by these significant economic and environmental concerns, alternative curing methods for PDMS elastomers have been proposed in recent years. For example, classical radical cross-linking, initiated by light or temperature-sensitive initiators, can be used to cure suitable PDMS derivatives.^13,15,16^ Especially photoinitiated thiol–ene chemistry has been applied for the preparation of PDMS elastomers and is useful in photochemical 3D printing applications.^17−19^ Tris(pentafluorophenyl)borane is applicable for borane hydrosilylation of poly(dimethyl-*co*-methylhydro)siloxane.^12^ Rieger and co-workers recently proposed a method employing silirene cross-linkers for photoactivated curing, avoiding any catalyst or additive during the cross-linking reaction.^10^ Although the system is compatible with different PDMS functionalities, it requires the laborious synthesis of silirene-linkers. Alternatively, polysilazanes can be used in combination with hydroxyl-functionalized PDMS, allowing network formation within 12 h at 60 °C, or autoxidation of hydrosilane-functionalized PDMS at temperatures above 220 °C.^20−22^ Various cross-linking procedures utilizing organic reactions such as Schiff-base formation,^3,23^ aza-Michael addition,^24,25^ or copper-free azide–alkyne cycloaddition^26,27^ have been reported as well, avoiding cost-intensive catalysts and enabling curing at ambient conditions. However, they partially require extensive PDMS functionalization and release additional side products during the reaction. Moreover, while ambient reaction conditions can be beneficial for certain processing methods, the lack of a curing trigger, such as temperature or light, necessitates two-component systems and complicates the implementation of the formulations for injection molding or 3D printing, for example.

**Figure 1:**
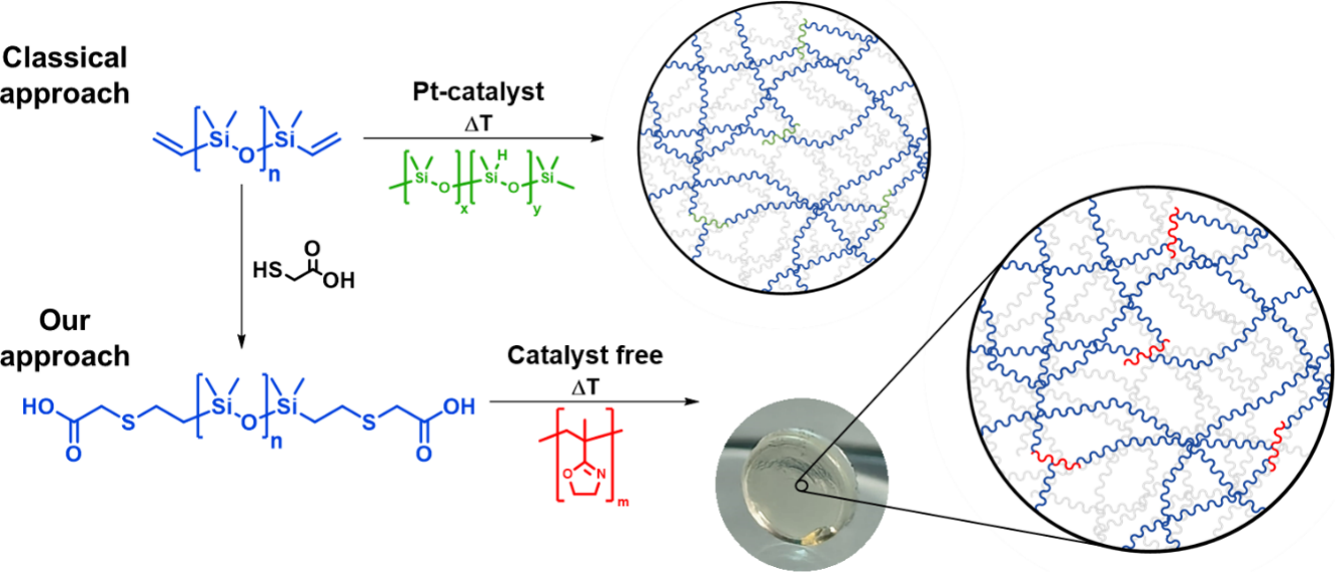
Schematic summary for the preparation of solvent-free PDMS elastomers via a metal-free PiPOx cross-linker-based curing approach in contrast to conventional Pt-catalyst-based systems.

Poly(2-isopropenyl-2-oxazoline) (PiPOx) is a highly promising biocompatible functional polymer bearing pendant 2-oxazoline rings.^28,29^ It can be synthesized by (controlled) radical polymerization and offers broad applicability in polymer science.^30−32^ Its capacity for postpolymerization modifications puts it in a unique spot for material development and design.^33^ Through a simple addition reaction with the pendant 2-oxazoline ring, a variety of functional groups can be reacted onto the polymer backbone in an atom-efficient manner without condensation products or the need for catalysts.^33^ Carboxylic acids, for instance, are reported to lead to a ring-opening reaction with good yields at elevated temperatures in polar aprotic solvents, providing a stable ester–amide linkage.^34^ This strategy was already employed manifold, labeling polymer particles or generating photoresponsive azo-polymers,^35,36^ as well as preparing functional materials based on PiPOx cross-linked polyester (hydro)gels or nanofiber networks.^37,38^ Herein, we present a novel method employing PiPOx cross-linkers for metal-free curing of PDMS elastomers, Figure 1, and study its suitability for processing by 3D printing. Additionally, since the biomedical field represents many of the major applications of PDMS elastomers, the biocompatibility of the novel formulations was investigated.

## Results and Discussion

### PDMS-PiPOx Reaction

The vinyl polymerization of the monomer 2-isopropenyl-2-oxazoline gives poly(2-isopropenyl-2-oxazoline) with pendant 2-oxazoline rings on each repeating unit.^29,32^ 2-Oxazolines are known to undergo a thermally triggered ring-opening addition reaction in the presence of carboxylic acids to give covalent ester–amide bonds.^33^ We, therefore, proposed that due to the thermally triggered nature of the ring-opening reaction, combined with the nonrequirement for catalysts and the absence of byproducts, PiPOx could provide a highly desirable cross-linker for PDMS elastomers.

**Figure 2:**
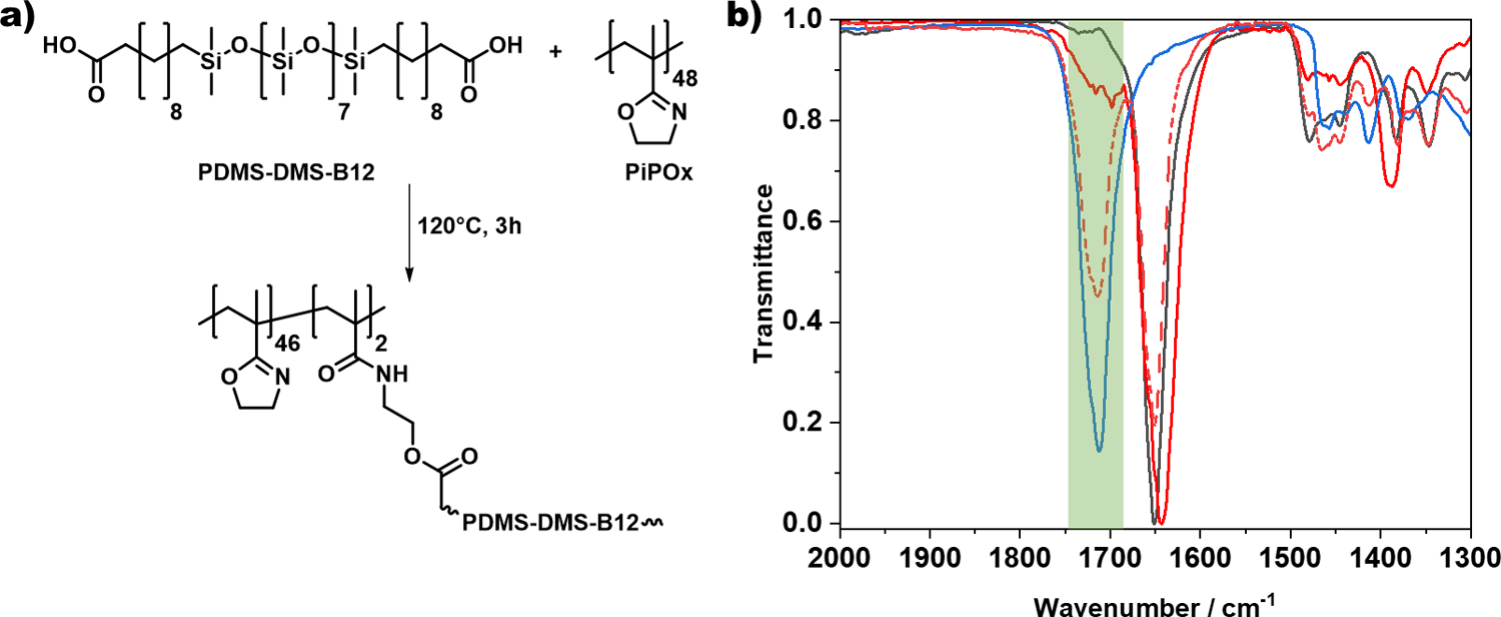
(a) Reaction scheme for the model reaction of PiPOx with PDMS-DMS-B12. (b) FTIR spectrum of the ring-opening reaction of PiPOx with PDMS-DMS-B12, showing the emerging signals corresponding to the formed ester–amide moiety. The pristine PiPOx is depicted in solid black, PDMS-DMS-B12 is in solid blue, the uncured formulation is in dashed red, and the functionalized copolymer is in solid red. The sample was reacted at 120 °C, cooled, and measured at room temperature.

A model reaction was performed (Figure 2a) to establish the general suitability of such an approach in which PiPOx (*M*_*n*_ = 5.3 kDa, *Đ* = 1.27) was combined with carboxylic acid-functionalized PDMS (PDMS-DMS-B12, *M*_*n*_ = 543 Da, *n* = 7 according to quantitative NMR spectroscopy) in a molar ratio of 0.5:10 of carboxylic acid functionalities to the pendant 2-oxazoline ring moieties. Separately, PiPOx was synthesized via highly controlled copper (0)-mediated reversible-deactivation radical polymerization (RDRP) to enable high reproducibility and command over the polymeric constituents (Figure S1). The mixture was heated to 120 °C in THF:DMF (4:1) (THF, tetrahydrofuran; DMF, *N,N*-dimethylformamide), and the reaction progress was observed via NMR and FTIR spectroscopies.^30,39^ In the FTIR spectrum (Figure 2b), the signals corresponding to the newly formed ester–amide moiety emerge at around 1700 and 1500 cm^–1^; however, overlapping bands at 1500 cm^–1^ impede a clear assignment. Meanwhile, in the ^1^H NMR spectrum, the appearance of a broad peak at around 3.3 ppm, associated with the ring-opened structure, emerges (Figure S2). Notably, the molecular weight of the PiPOx constituent appeared to strongly influence the reactivity, with a decreasing conversion observed with increasing *M*_w_.

### Platinum- and Solvent-Free Curing of PDMS

**Figure 3:**
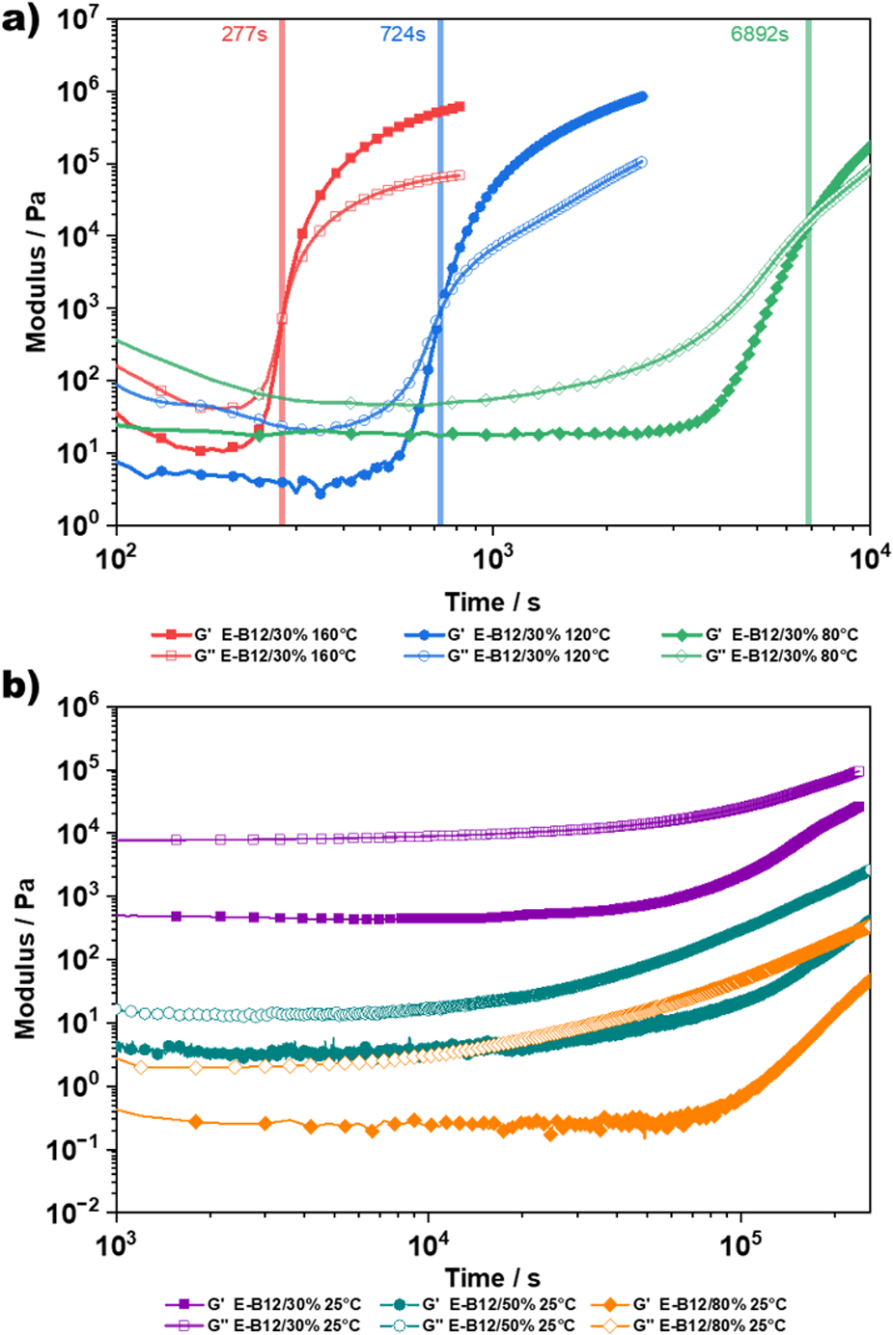
(a) Investigation of the curing kinetics of PDMS-DMS-B12 cross-linked with PiPOx on a plate–plate rheometer at 160, 120, and 80 °C, showing a clear dependence of the curing time on temperature. (b) Determination of the storage stability of E-B12/30%, E-B12/50%, and E-B12/80% formulations at room temperature.

In the next step, metal-free and solvent-free curing of PDMS-DMS-B12 was carried out by direct mixing of PiPOx with 15 equiv of the PDMS diacid, resulting in a ratio of 3:10 acid to 2-oxazoline moieties (E-B12/30%). Although PiPOx was immiscible with PDMS-DMS-B12, homogeneous elastomer pellets could be obtained after thoroughly mixing the PiPOx/PDMS-DMS-B12 mixture by hand with a spatula and curing it subsequently. A photograph of the prepared pellet can be seen in Figure S3. The formulation was further reacted on a plate–plate rheometer at various temperatures to investigate the reaction kinetics. Rapid curing in 4.5 min can be observed upon heating at 160 °C, indicated by the crossover point of the storage modulus (*G*′) and loss modulus (*G*″) (Figure 3a). A decrease in the reaction temperature down to 120 or 80 °C, in turn, increases the curing time required to around 12 min or 2 h, respectively. Although no crossover point can be observed even after 72 h at room temperature (RT), a slight onset of increasing moduli can be seen after around 12 h, indicating a stable formulation during storage at 25 °C for at least 12 h for each formulation of the E-B12 series (Figure 3b).

Subsequently, a series of PiPOx-PDMS elastomers based on PDMS-DMS-B12 (E-B12) was synthesized and cast in circular Teflon molds. The elastomers were prepared targeting an increasing degree of ring-opening of the 2-oxazoline rings, corresponding to the molar ratio of carboxylic acid functionalities on the PDMS constituents to the pendant 2-oxazoline ring moieties of the PiPOx constituents (3:10 in E-B12/30%, 5:10 in E-B12/50%, and 8:10 in E-B12/80%, Table 1). FTIR measurements of the prepared elastomer pellets showed a successful ring-opening reaction of the 2-oxazoline rings and incorporation of PiPOx in the elastomer structure (Figure S4). The mechanical properties of the elastomers were established by rheological measurements, measuring both frequency and amplitude sweeps (Figure S5). From the frequency sweeps, an apparent rubberlike behavior can be discerned with a plateau stretching over the frequency range from 10^–1^ to 10^2^ rad*s^–1^. Interestingly, a higher degree of targeted ring-opening and, thus, a targeted increase in the degree of cross-linking does not result in the expected increase in moduli. This may be explained by steric reasons, hindering higher degrees of ring-opening on the PiPOx backbone and therefore resulting in a lower effective degree of cross-linking or possibly due to the significantly increased weight content of PDMS for this series from 60% for E-B12/30% to over 70% for E-B12/50% and 80% for E-B12/80%. The solid content of produced elastomers was also determined by washing the pellets in CH_2_Cl_2_ over 24h, followed by drying on air for 48h, and showed gel fractions above 90% for all three formulations, Table S2.

**Table 1:** Summary of Prepared *PDMS-PiPOx* Formulations^a^

formulation	degree of prepolymerization/%	PiPOx constituent	PDMS constituent	degree of RO*/%	weight content PDMS constituent (including end-groups)/%
E-B12/30%		RDRP-PiPOx	DMS-B12	30	60
E-B12/50%		RDRP-PiPOx	DMS-B12	50	71
E-B12/80%		RDRP-PiPOx	DMS-B12	80	80
E-B25/30%	7.5	RDRP-PiPOx	DMS-B25	30	93
E-B25/50%	7.5	RDRP-PiPOx	DMS-B25	50	96
E-B25/80%	7.5	RDRP-PiPOx	DMS-B25	80	98
E-Syl/50%	7.5	FRP-PiPOx	Syl-PDMS	50	95
E-Syl/50%-Aerosil	7.5	FRP-PiPOx	Syl-PDMS	50	87

a RO corresponds to the targeted degree of ring-opening reactions of the pendant 2-oxazoline rings on PiPOx and hence the molar ratio of carboxylic acid functionalities to the pendant 2-oxazoline ring moieties. E-Syl/50%-Aerosil includes 10 wt % powdered filler material Aerosil R 106. RDRP: reversible-deactivation radical polymerization; FRP: free-radical polymerization.

### Curing of PDMS via a Prepolymer Approach

**Figure 4:**
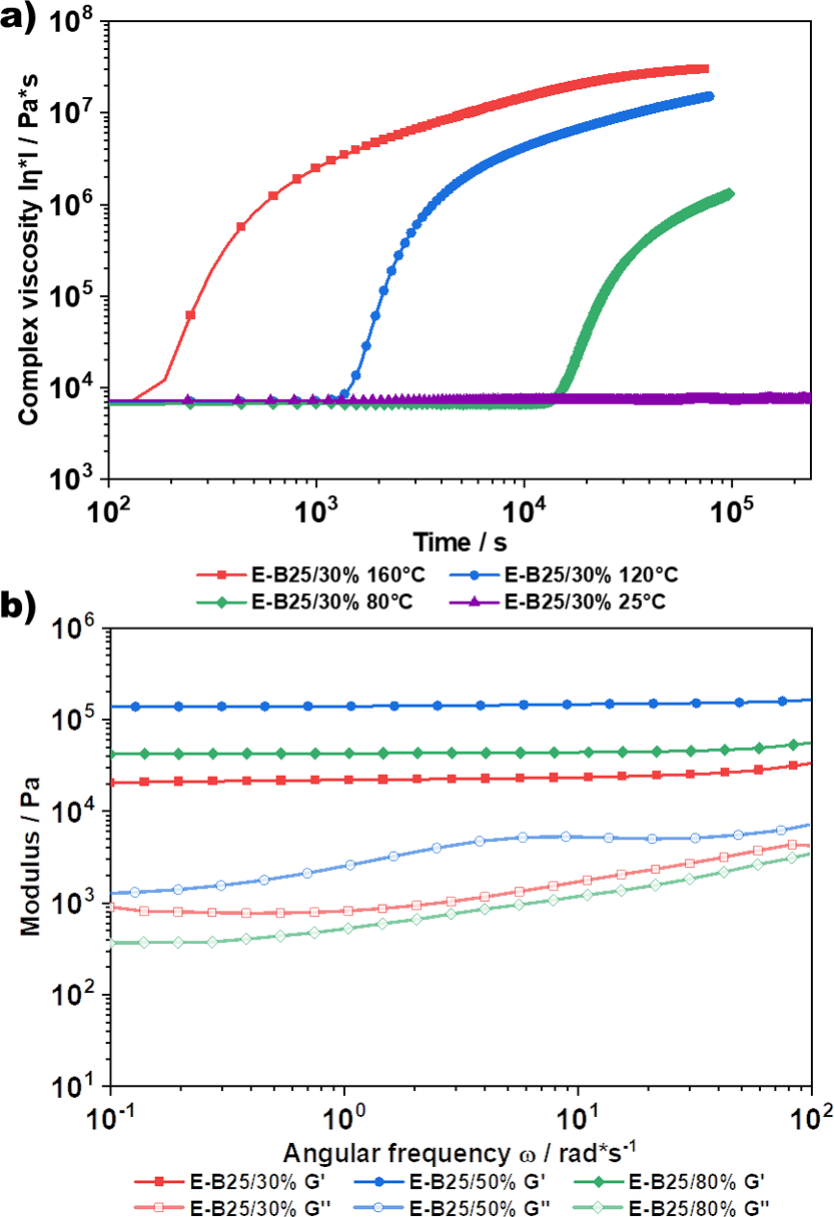
(a) Investigation of the curing kinetics of PDMS-DMS-B25 cross-linked with PiPOx via a prepolymer approach. The increase in complex viscosity was monitored on a plate–plate rheometer at 160, 120, 80, and 25 °C, showing a clear dependence of the curing time on temperature, indicated by the increase in viscosity. (b) Frequency sweeps of elastomers E-B25/30%, E-B25/50%, and E-B25/80%, showing a clear rubberlike behavior over the frequency range 0.1–100 rad*s^–1^.

The mechanically brittle characteristics of the E-B12 elastomer series were mainly attributed to the short chain length of the PDMS constituent DMS-B12.^40^ Therefore, to increase the elasticity, a second series of elastomers (E-B25) with a longer PDMS chain, based on PDMS-DMS-B25 (*M*_*n*_ = 7030 Da, *n* = 94 according to quantitative NMR spectroscopy), was prepared (Table 1). However, in contrast to the elastomer series E-B12, direct solvent-free curing resulted in the demixing of the immiscible constituents during the curing process and led to the formation of an inhomogeneous mixture. Hence, a prepolymer approach was developed, reacting PiPOx first with 4 equiv of the PDMS-DMS-B25 diacid in THF:DMF (4:1) at 120 °C for 3 h and keeping the mixture below the gel point. After the viscous prepolymer was mixed with additional PDMS-DMS-B25, the solvent was evaporated to achieve the final completely miscible formulation, and the elastomers were cured in the oven. The reaction kinetics were again studied by rheology (Figure 4a). For the prepolymer series, the onset of curing was monitored by the increase in viscosity since *G*″ was already below *G*′ at the beginning of the measurement, possibly due to the partial preliminary cross-linking below the gel point. While an onset of curing can already be observed after around 3 min at 160 °C, generally longer curing times were observed in comparison to the E-B12 elastomers as a result of the higher *M*_*n*_ of the PDMS chains. Similarly to the E-B12 series, curing times were slower at lower temperatures, and no significant change in viscosity can be observed even after 3 days at 25 °C, further demonstrating the benchtop stability of this one-component formulation at RT.

**Figure 5:**
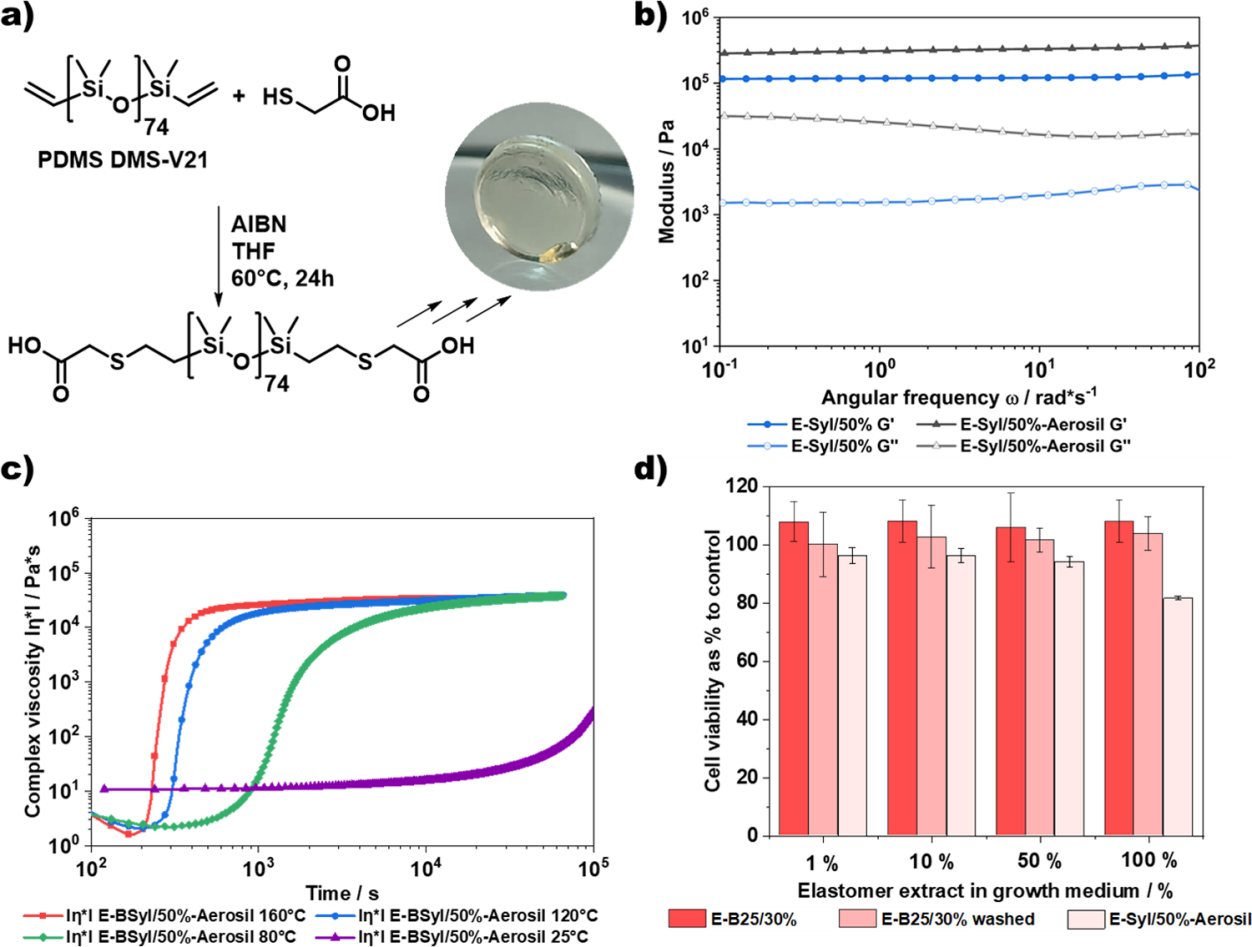
(a) Schematic representation of the synthesis of carboxylic acid-functionalized PDMS via simple thiol–ene chemistry and subsequent curing. (b) Frequency sweeps of elastomers E-Syl/50% and E-Syl/50%-Aerosil, showing a clear rubberlike behavior over the frequency range 10^–1^–10^2^ rad*s^–1^ as well as the influence of the filler content on the mechanical characteristics. (c) Investigation of the curing kinetics of the E-Syl/50%-Aerosil formulation. The increase in complex viscosity was monitored on a plate–plate rheometer at 160, 120, 80, and 25 °C, showing a clear dependence of the curing time on temperature indicated by the increase in viscosity. (d) In vitro cell cytotoxicity of E-B25/30% before and after washing in CH_2_Cl_2_, as well as E-Syl/50%-Aerosil using extracts of the elastomers and evaluated via MTT cell viability assays on mouse 3T3 fibroblasts.

The cured elastomers were further investigated by FTIR spectroscopy and micro-FTIR in combination with XPS, showing a successful ring-opening reaction of the 2-oxazoline rings and incorporation of PiPOx in the elastomer structure (Figures S6b, S7–S9, and Table S1). The deconvoluted XPS peaks clearly confirm the presence of PDMS (C, Si, and O detection trace) and PiPOx (C, O, and N detection trace), both before and after washing with CH_2_Cl_2_ with no significant difference, underlined by the quantification in Table S1. Overall, the bulk mass of surface material could be ascribed to PDMS, with around 22% for Si 2p and matching 44 and 20% for the C 1s (C–Si) and O 1s (Si–O) peaks, respectively. Residues of the Teflon mold could be detected as well, based on the C and F detection trace; however, these were reduced upon washing. Given the theoretical elemental composition of PiPOx of 12.7% N, 65.5% C, 14.5% O, and 8.2% H, a N content of 0.3 to 0.4% according to the XPS analysis would correspond to approximately 3–5 mol % of PiPOx in the PDMS-PiPOx elastomers, which corresponds approximately to the feed ratio. Rheological measurements were also performed to establish the mechanical characterization (Figures 4b and S6a). The frequency sweeps show a clear rubberlike behavior over the frequency range from 0.1 to 100 rad*s^–1^, indicated by the plateau modulus. As expected the moduli increase from E-B25/30% to E-B25/50%, corresponding to higher ring-opening and hence the degree of cross-linking. Interestingly, the higher ratio of acid to 2-oxazoline rings for E-B25/80% does not conform to this expected trend of increasing moduli. This may be explained by steric hindrance of the 2-oxazoline ring-opening reaction at these higher conversions and a potentially lower stoichiometric cross-linking efficiency, effectively decreasing the apparent degree of cross-linking and possibly resulting in dangling chain ends which lower the modulus. The gel fractions of the materials are summarized in Table S3. Overall, softer materials were prepared with the longer PDMS diacid PDMS-DMS-B25 compared to the E-B12 series, shown by the decrease in the modulus from roughly 10^7^ to 10^5^ Pa. As a significant application for both PiPOx and PDMS biomaterials, the biocompatibility and cytotoxicity of the materials were investigated via MTT assays. No cytotoxic effect on the cell growth could be observed upon incubation of 3T3 mice fibroblast cells with extracts from E-B25/30%, both before and after washing, as well as upon direct contact with the elastomers according to ISO 10993 (Figures 5d and S10).

### Vinyl PDMS-Based Systems

Sylgard is one of the most widely used commercial PDMS curing systems and involves a two-component procedure containing a Pt catalyst for hydrosilylation. The system combines a divinyl PDMS with a multihydride cross-linker (Figure 1). Hence, to adapt the approach to widely available vinyl-based systems, a divinyl-terminated PDMS (100 cSt) was functionalized with thioglycolic acid via a simple and scalable thiol–ene reaction (Figures 5a and S11). In addition, PiPOx polymerized via free-radical polymerization (FRP-PiPOx, *M*_w_ = 4.5 kDa, *Đ* = 4.7, Figure S12) was prepared to allow for a larger-scale preparation at the expense of dispersity control. This step further eliminates the use of metal catalysts that were necessary during controlled radical polymerization. The two components were then formulated according to the prepolymer approach developed for the E-B25 series and cured at 120 °C with 50% ring-opening of the 2-oxazoline rings (E-Syl/50%). Moreover, as commercial silicone elastomers are usually prepared as composites, the incorporation of Aerosil R 106 was tested by simple mixing of the PDMS-PiPOx formulation with the powdered filler material, targeting a filler content of 10 wt % (E-Syl/50%-Aerosil).

The gel fractions of both elastomers were determined as previously described by washing with CH_2_Cl_2_ and subsequent drying (Table S4), and the materials were further characterized by rheological means (Figures 5b and S13). From the frequency sweeps, a clear comparison between E-Syl/50% and E-B25/50% can be made, showing only marginal differences and hence no adverse effect of the FRP approach for the PiPOx moiety in E-Syl/50%, while the incorporation of 10 wt % Aerosil R 106 leads to the expected increase in the mechanical modulus. In addition, tensile tests were performed, and test specimens of E-Syl/50%-Aerosil were compared to Sylgard-184 (Figure S14). With a maximum stress of around 15–20 MPa at approximately 30% strain, the E-Syl/50%-Aerosil formulation is considerably weaker compared to Sylgard-184 and hence will need further optimization to compete with commercial products.

The reaction kinetics of the E-Syl/50%-Aerosil formulation was studied as described for the E-B25/50% formulation, observing the onset of curing via the increase in viscosity. Similar to the E-B25/50% formulation (Figure 4a), an onset of curing can already be observed after around 3 min at 160 °C (Figure 5c). The effect of longer curing times when compared to the E-B12 elastomers remains; however, quicker curing of the E-Syl/50%-Aerosil formulation is apparent by the sharp increase in viscosity. Similar to the E-B12 and E-B25 series, curing times were slower at lower temperatures, again demonstrating the benchtop stability of these one-component formulations at room temperature. The biocompatibility and cytotoxicity of the E-Syl/50%-Aerosil formulation intended for 3D printing were tested, analogous to E-B25/30%. Again, no cytotoxic effect on the cell growth of 3T3 mouse fibroblast cells could be observed upon incubation with extracts from E-Syl/50%-Aerosil. Measurements regarding direct contact toxicity were performed according to ISO 10993 and did not show any cytotoxicity as well (Figures 5d and S10).

### 3D Printing

**Figure 6:**
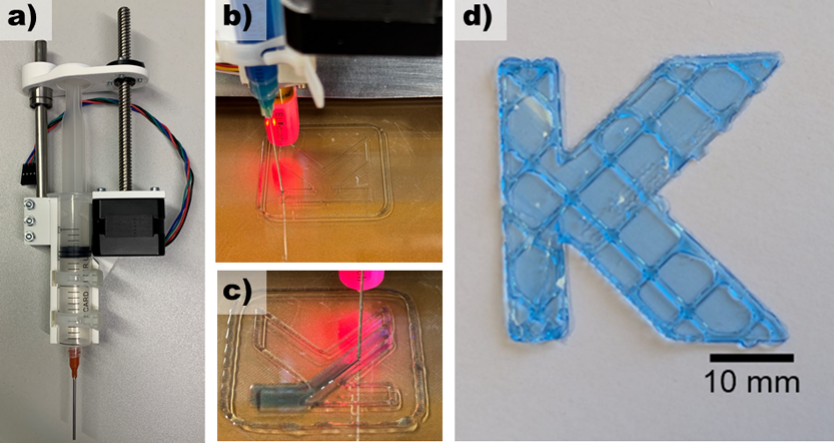
Printhead (a) used for direct ink writing of the elastomer formulation E-Syl/50%-Aerosil along with images taken during the printing process (b, c) as well as a printed specimen in the letter K (d). The formulation was colored blue for better visualization.

Finally, the E-Syl/50%-Aerosil formulation was tested for its suitability as a 3D printing ink for direct ink writing. Since 3D printing requires much larger amounts of materials, FRP-PiPOx was used as it is easily accessible and scalable compared to RDRP-PiPOx. Although addition of the Aerosil-filler was not necessary to enable a printable formulation, the addition of the filler increases the viscosity of the formulation, simplifying the printing process. First, the material was dyed light blue with a silicone pigment for better visualization. The material was then transferred to a 3 mL syringe and evacuated in a vacuum chamber for 20 min to remove any trapped air. After assembly of the syringe-based print head, which was adapted with a IC blunt needle with an inner diameter of 0.8 mm, the material was printed on a borosilicate glass plate with a layer thickness of 0.4 mm using a print speed of 20 mm*s^–1^ (Figure 6a–c). Each layer was cured by irradiation with a 2000 W halogen lamp for 90 s and a distance of 30 mm from the printed layer, reaching the required curing temperatures on the surface (Figure S15) and finally cut from the glass support after cooling down. As can be seen in Figure 6d, a clear and detailed letter K could be printed with a resolution of approximately 1 mm, highlighting the suitability of the developed formulation for manufacturing via 3D printing. Furthermore, the benchtop stability of the formulation allowed printing even after 7 days of standing.

## Conclusions

In conclusion, an innovative solution for the metal- and solvent-free curing of PDMS elastomers has been reported. Based on the atom-efficient reaction of pendant 2-oxazoline rings of PiPOx with carboxylic acids, a unique cross-linker chemistry for PDMS elastomers was developed and employed to cure PDMS diacids in a thermally triggered reaction in the absence of catalysts or solvents. While low-molecular-weight PDMS could be cured directly, a two-step prepolymer approach was developed to overcome the lower reactivity and demixing of higher-molecular-weight PDMS diacids. Furthermore, the simple conversion of divinyl PDMS to the required carboxylic acids meant that the approach could be easily extended to widely available silicone elastomer systems. The oxazoline curing method represents a bench stable, completely metal-free, solvent-free, biocompatible, and atom-efficient route to cured PDMS elastomers that does not elute volatiles. These properties, combined with the temperature-triggered curing, mean that this method facilitates advanced manufacturing by direct ink writing and, in the future, could potentially be expanded to other industrially important extrusion-based methods such as injection molding.

## Materials & Methods

PDMS-DMS-B12 [(carboxydecyl)-terminated polydimethylsiloxane, 15–30 cSt] and PDMS-DMS-B25 [(carboxydecyl)-terminated polydimethylsiloxane, 450–550 cSt] were purchased from Gelest and a vinyl-terminated PDMS (100 cSt) from abcr. Dichloromethane (CH_2_Cl_2_) and DMF of HPLC grade were obtained from Fisher Chemical, LiBr was obtained from Alfa Aesar, and chloroform, diethyl ether (Et_2_O), THF, MgSO_4_, and DMF were purchased from VWR. 3-(4,5-Dimethyldiazol-2-yl)-2,5-diphenyltetrazolium bromide (MTT) was bought from Calbiochem, Dulbecco’s modified Eagle medium (DMEM) and fetal bovine serum (FBS) were purchased from Gibco, DMF-*d*_7_ was purchased from Deutero, and Aerosil R 106 was purchased from Evonik. *N,N*-Dimethylacetamide (DMAc), 2-isopropenyl-2-oxazoline (iPOx), CuCl, CuCl_2_, tris(2-pyridylmethyl)amine (TPMA), 2-chloropropionitrile (CPN), 2,2′-azobis(2-methylpropionitrile) (AIBN), and thioglycolic acid were purchased from Sigma-Aldrich. iPOx was distilled under reduced pressure prior to RDRP. For FRP, the monomer was used as received. Dialysis was performed with a no.6 dialysis tubing from Spectrum Laboratories with a molecular weight cutoff (MWCO) of 1 kDa.

Size exclusion chromatography (SEC) was carried out on two different instruments. On the one hand, a Viscothek GPCmax instrument from Malvern, equipped with a PFG column from Polymer Standard Service GmbH (300 × 8 mm, 5 μm particle size) and run with DMF containing 10 mM LiBr as the mobile phase, was used. The samples were prepared as a 10 mg*mL^–1^ solution, filtered through a 0.2 μm PTFE syringe filter prior to injection, and eluted at a flow rate of 0.75 mL*min^–1^ at 60 °C. The system was calibrated with polystyrene standards from the Polymer Standard Service GmbH using a conventional calibration of the refractive index detector. On the other hand, an SEC system consisting of Shimadzu LC–20, a Shimadzu refractive index detector, and two PPS PFG 5 μm columns or PSS GRAM 5 μm columns from Polymer Standard Service GmbH (300 mm × 8 mm) at 25 °C were employed. DMAc with an addition of 0.1 wt % LiBr was used as an eluent at a flow rate of 1 mL*min^–1^, and samples were injected with a concentration of 1 mg*mL^–1^. Poly(methyl methacrylate) standards from Polymer Standard Services GmbH were used for calibration.

Infrared (IR) spectra were recorded with a PerkinElmer 100 Series FTIR spectrometer equipped with ATR using a scan number of 128 as well as on a Nicolet 8700 FTIR spectrometer from Thermo scientific equipped with a Nicolet Continuum microscope and a germanium ATR crystal using 64 scans and a resolution of 4 cm^–1^.

Nuclear magnetic resonance (NMR) measurements were performed on a Bruker Avance III 300 MHz spectrometer in deuterated solvents at 25 °C, referenced to the respective internal solvent signal.

XPS signals were recorded using a Thermo Scientific Nexsa G2 surface analysis system equipped with a microfocused, monochromatic Al Ka X-ray source (1486.68 eV). An X-ray beam of 400 mm size was used. The spectra were acquired in the constant analyzer energy mode with a pass energy of 200 eV for the survey. Narrow regions were collected using a pass energy of 50 eV. Charge compensation was achieved with the system dual beam flood gun. The Thermo Scientific Avantage software, version 6.7.0, was used for digital acquisition and data processing. Spectral calibration was determined by using the automated calibration routine and the internal Au, Ag, and Cu standards supplied with the K-Alpha system. The surface compositions (atomic %) were determined by considering the integrated peak areas of the detected atoms and the respective sensitivity factors. The fractional concentration of a particular element A was computed using
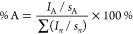
where *I*_*n*_ and *s*_*n*_ are the integrated peak areas and the Scofield sensitivity factors corrected for the analyzer transmission, respectively.

The elastomer samples for rheological characterization and gel fractions were cured in a circular Teflon mold in a drying and heating chamber from Binder at 120 °C. Rheological characterizations were conducted on an Anton Paar MCR 502 rheometer with a plate–plate geometry (8, 10, and 25 mm diameters) through stress-controlled oscillatory tests at 24 °C. Amplitude sweeps were performed with a constant frequency of 1 rad*s^–1^ and an increasing strain rate γ from 10^–3^ % to 10° % for E-B12 and 10^–2^ % to 10^2^ % for E-B25 and E-Syl. Subsequently, frequency sweeps at a constant rate of deformation of γ = 1 × 10^–2^ % for E-B12 and of γ = 1% for E-B25 and E-Syl were conducted. The reaction kinetics was directly investigated by measuring the freshly prepared formulations on the heated rheometer plate at the respective temperatures (room temperature, 80, 120, and 160 °C). The gel fractions of the elastomer pellets were determined by washing the pellets with CH_2_Cl_2_ over 24 h. After separation of the solid fraction by decanting or filtration and subsequent drying on air at RT for 48 h, the weight loss of pellets was determined and the gel fraction was calculated. Tensile tests were performed on a TA Instruments DMA Q800 with an increase in the applied normal force of 0.25 N per 60 s and a sample geometry of width = 40 mm, length = 100 mm, and thickness = 2 mm.

Cytotoxicity of elastomers was tested using extracts according to ISO 10993 and direct contact cytotoxicity. Extracts from two types of elastomers were obtained by extraction into growth medium (DMEM + 10% FBS) for 24 h. The extract thus obtained was labeled as 100% and was subsequently diluted with growth medium to 50, 10, and 1% solutions. The pure extracts and their dilutions were then added to 3T3 fibroblasts that grew on a 96-well tissue culture plate at a concentration of 5 × 10^3^ cells per well. After 24 h of incubation, quantification of living cells was performed using the MTT test. Extracts from wells were replaced by 100 μL of MTT at a concentration of 0.5 mg*mL^–1^ in growth media (DMEM + 10% FBS) and incubated for 3 h. Afterward, MTT was removed, and 100 μL of dimethyl sulfoxide was added to the wells to extract the water-insoluble formazan formed by living cells. The absorbance was measured via a plate reader at 595 nm (Multiscan FC, Thermofisher). For contact cytotoxicity, cells were seeded in the 24-well tissue culture plate at a concentration of 50 × 104 cells per well and incubated overnight. The next day, pieces of elastomers covering 10% of the well surface were placed in triplicate into the cell containing wells and incubated for 24 h, followed by an MTT assay as described above.

### PiPOx RDRP Synthesis (RDRP-PiPOx)

Copper(0)-mediated RDRP of 2-isopropenyl-2-oxazoline was performed according to literature procedures.^41,42^ Briefly, the polymerization was set up with 2-isopropenyl-2-oxazoline (iPOx):CuCl:CPN:CuCl_2_:TPMA in molar ratios of 50:1:1:0.5:1.5. First, a mixture of TPMA, CuCl, and CuCl_2_ was weighed in and deoxygenated via 3–5 vacuum/argone cycles. The mixture was dissolved in degassed, aqueous NaCl (0.67 M, 1 mL), and iPOx was added, resulting in a turbid, light-green reaction solution. Subsequently, the CPN initiator was introduced, and the reaction was stirred at RT for 24 h. After dilution of the reaction solution with 4 mL of distilled water, the product was dialyzed (1 kDa MWCO) for 24 h against water and ethanol, with three changes of dialysis medium. The final product was freeze-dried and stored in a desiccator at 5 °C.

^1^H NMR (300 MHz, DMF-*d*_7_, δ): 0.89–1.37 (m, 3H), 1.62–2.08 (m, 2H), 3.73 (br, 2H), 4.22 ppm (br, 2H).

*M*_w_ = 5.3 kDa, *Đ* = 1.27.

### PiPOx FRP Synthesis (FRP-PiPOx)

FRP of iPOx was performed under inert conditions. iPOx (4.72 mL, 5.00 g, 45 mmol, 33 equiv) was transferred into a Schlenk tube along with 222 mg of AIBN (1.4 mmol, 1 equiv), dried under vacuum, subsequently flushed with Ar, and diluted with 3 mL of chloroform (dried over 3 Å molecular sieves). The reaction was stirred for 3 h at 80 °C and afterward cooled to RT. The product was precipitated twice in diethyl ether, isolated by decanting, dried, and stored in a desiccator at 5 °C (3.29 g, 66%).

^1^H NMR (300 MHz, DMF-*d*_7_, δ): 0.95–1.35 (m, 3H), 1.66–2.10 (m, 2H), 3.72 (br, 2H), 4.22 ppm (br, 2H).

*M*_w_ = 4.5 kDa, *Đ* = 4.7.

### Sylgard PDMS Functionalization (Syl-PDMS)

Carboxylic acid-functionalized PDMS derived from divinyl-terminated PDMS (100 cSt) was prepared via a simple thiol–ene addition reaction, denoted Syl-PDMS. To this end, 100 g of vinyl-terminated PDMS (19 mmol, 1 equiv) was dissolved in 200 mL of THF and mixed with 0.5 g of AIBN (3 mmol, 0.5 wt %) and 4.02 mL of thioglycolic acid (58 mmol, 3 equiv). After degassing the reaction solution for 20 min with Ar, it was heated under stirring for 24 h at 60 °C. Subsequently, the solvent was removed by rotary evaporation, taken up again in 500 mL of CH_2_Cl_2_, and washed five times, each with 150 mL of brine. The combined aqueous phase was extracted once with 150 mL of CH_2_Cl_2_, and the combined organic phase was then dried over MgSO_4_, filtered, and evaporated again via rotary evaporation. The product was obtained as a clear highly viscous oil after drying in a desiccator for 24h (89 g, 86%).

^1^H NMR (300 MHz, CDCl_3_, δ): 0.23 – (−0.07) (m, 210H), 0.85–0.99 (m, 2H), 2.63–2.79 (m, 2H), 3.27 ppm (s, 2H).

### Preparation of Elastomers E-B12

PDMS-PiPOx elastomers based on PDMS-DMS-B12 (*M*_*n*_ = 543 Da, *n* = 7 according to quantitative NMR spectroscopy) were prepared with varying percentages of targeted degree of ring-opening, namely, 30, 50, and 80%. In general, PDMS-DMS-B12 and PiPOx (synthesized by RDRP, *M*_*n*_ = 5.3 kDa, *Đ* = 1.27) were weighed into a vial and thoroughly mixed with a spatula by hand. After transfer of the formulation into circular Teflon molds, the material was cured in an oven at 120 °C for 24 h.

Exemplary for E-B12/50%, 756.7 mg of PDMS-DMS-B12 (1.35 mmol, 0.5 equiv) was mixed with 300 mg of PiPOx (2.70 mmol of repeating units, 1 equiv), and the viscous paste was filled into circular Teflon molds. After the formulation was cured at 120 °C for 24 h, the pellets were cooled to RT and stored under air at RT.

### Preparation of Elastomers E-B25

To enable the formation of elastomers based on the longer PDMS diacid PDMS-DMS-B25 (*M*_*n*_ = 7030 Da, *n* = 94 according to quantitative NMR spectroscopy) with PiPOx (synthesized by RDRP, *M*_*n*_ = 5.3 kDa, *Đ* = 1.27), a prepolymerization approach was followed, in contrast to the direct curing for the E-B12 series. First, PDMS-DMS-B25 was reacted with PiPOx in a solvent mixture of THF:DMF (4:1) at 120 °C for 3 h with a targeted degree of ring-opening of 7.5%. After addition of PDMS-DMS-B25 to achieve the final targeted degree of ring-opening, the reaction mixture was evaporated to complete dryness, and the solvent-free formulation was transferred in circular Teflon molds and cured for 24 h at 120 °C.

For elastomer E-B25/50%, the procedure is as follows. 40.2 mg of PiPOx (0.365 mmol, 1 equiv) and 192.1 mg of PDMS-DMS-B25 (0.027 mmol, 0.075 equiv) were dissolved in a solvent mixture of 1.8 mL of THF and 0.45 mL of DMF. The reaction vessel was tightly closed and stirred at 120 °C for 3 h. Next, 895.4 mg of PDMS-DMS-B25 (0.105 mmol, 0.425 equiv) was added, the solvent mixture was evaporated, and the completely dry, solvent-free elastomer formulation was poured into circular Teflon molds. After curing at 120 °C for 24 h, the pellets were cooled to RT, removed from the molds, and stored under air.

### Preparation of Elastomers E-Syl

The elastomer formulations were prepared analogous to the E-B25 series. 207.8 mg carboxylic acid-functionalized Syl-PDMS (0.039 mmol 0.075 eq., *M*_*n*_ = 5370 Da, *n* = 70 according to quantitative NMR spectroscopy) was reacted with 56.8 mg of PiPOx (0.516 mmol, 1 equiv, synthesized by FRP, *M*_*n*_ = 4.5 kDa, *Đ* = 4.7) in a prepolymerization step at 120 °C for 3 h in a solvent mixture of THF:DMF (4:1, 1.8 mL:0.45 mL), targeting a degree of ring-opening of 7.5%. After addition of the remaining carboxylic acid-functionalized Syl-PDMS (957.5 mg, 0.178 mmol, 42.5 equiv), the solvent was evaporated. Filler material Aerosil R 106 was added portionwise under constant stirring with a content of 10–14 wt % for E-Syl/50%-Aerosil. Finally, the formulations were poured into circular Teflon molds and cured for 24 h at 120 °C. After cooling at RT, the pellets were stored under air.

### 3D Printing

Before printing, the material was dyed light blue using a silicone pigment dye and then transferred to a 3 mL syringe with a Luer Lock connector. To remove any excess air that might have been trapped in the system, the open syringe was placed in a vacuum chamber and degassed for 20 min.

The printer used in this study is based on the Ratrig V-Core Pro mechanical kit (Ratrig, Portugal) in which the standard thermoplastic extruder was replaced by a syringe extruder that was actuated by a T8 lead-screw. Additionally, a 2000 W halogen lamp was mounted to the *x*-axis, where it did not interfere with the extruder. The printer is controlled by a Duet3d Duet Ethernet control board with a Duex5 (Duet3D Ltd., Peterborough, United Kingdom) extension board running Reprap-Firmware. All G-codes used in this study were sliced using Prusaslicer (Prusa Research a.s., Prague, Czech Republic)

The material was then printed on a borosilicate glass plate by using a 0.8 mm ID blunt needle with a layer height of 0.4 mm and a print speed of 20 mm*s^−1^. To demonstrate the ability to generate thin structures, a low infill percentage of 5% was chosen. After each layer, the deposited material was partially cured using a 2000 W halogen lamp for 90 s at a distance of 30 mm from the printed layer. The part was printed in an unenclosed printer, and apart from the halogen lamp, no additional heating was used. After the final layer was printed, the print was left to cool for 30 min and then removed from the build-plate with a sharp blade.

The temperature change under irradiation during curing was observed by embedding a temperature sensor in a precast pellet of E-Syl/50%-Aerosil (*d* = 26 mm, *h* = 10 mm) at a depth of 4 mm below the upper surface. A Type K thermocouple, connected to a Testo 176T4 temperature logger, was used, and the exposed cable was isolated with four layers of braided glass fiber hose and shielded with aluminum foil to minimize the influence of direct irradiation on the sensor. All distances were maintained as if the printer had just completed the top layer of the precast pellet. The measurement was simultaneously started with the heating of the specimen’s surface.

## Supporting Information Available

^1^H NMR spectrum; photographs of prepared elastomer pellets; FTIR spectrum of elastomer E-B12/50% at 25 °C; amplitude sweeps; micro-ATR spectrum; deconvolution of the XPS peaks for nitrogen, silicon, carbon, and oxygen of E-B25/30%; in vitro contact cytotoxicity of elastomers; tensile tests; temperature profile during 3D printing and curing; apparent surface chemical composition of E-B25/30%; and gel fraction of the PiPOx-PDMS elastomers (PDF)

## References

[ref1] Brook M. A. Silicon in organic, organometallic, and polymer chemistry; A Wiley-Interscience Publication, 2000.

[ref2] Mark J. E.; Schaefer D. W.; Lin G. The polysiloxanes; Oxford University Press, 2020.

[ref3] Bui R.; Brook M. A. Catalyst Free Silicone Sealants That Cure Underwater. Adv. Funct. Mater. 2020, 30 (23), 2000737 10.1002/adfm.202000737.

[ref4] Chiaula V.; Mazurek P.; Eiler J.; Nielsen A. C.; Skov A. L. Glycerol-silicone adhesives with excellent fluid handling and mechanical properties for advanced wound care applications. Int. J. Adhes. Adhes. 2020, 102, 102667 10.1016/j.ijadhadh.2020.102667.

[ref5] Tugui C.; Stiubianu G. T.; Serbulea M. S.; Cazacu M. Silicone dielectric elastomers optimized by crosslinking pattern – a simple approach to high-performance actuators. Polym. Chem. 2020, 11 (19), 3271–3284. 10.1039/D0PY00223B.

[ref6] Kuah H. X.; Loh X. J. Silicones: The Future for Beauty and Everyday Care. In Polymers for Personal Care Products and Cosmetics; Royal Society of Chemistry, 2016.

[ref7] Ajvazi E.; Bauer F.; Strasser P.; Brüggemann O.; Preuer R.; Kracalik M.; Hild S.; Abbasi M.; Graz I.; Teasdale I. Inorganic Bottlebrush and Comb Polymers as a Platform for Supersoft, Solvent-Free Elastomers. ACS Polymers Au 2024, 4 (1), 56–65. 10.1021/acspolymersau.3c00043. 38371734 PMC10870749

[ref8] Silicone Market Size, Share & Trends Analysis Report, 2030. https://www.grandviewresearch.com/industry-analysis/silicone-market (accessed Apr 29, 2024).

[ref9] Marmo A. C.; Grunlan M. A. Biomedical Silicones: Leveraging Additive Strategies to Propel Modern Utility. ACS Macro Lett. 2023, 12 (2), 172–182. 10.1021/acsmacrolett.2c00701. 36669481 PMC10848296

[ref10] Nobis M.; Futter J.; Moxter M.; Inoue S.; Rieger B. Photo-Activity of Silacyclopropenes and their Application in Metal-Free Curing of Silicones. ChemSusChem 2023, 16 (3), e202201957 10.1002/cssc.202201957. 36445812 PMC10107829

[ref11] Friedman D.; Tina M.; Steve O. The Role of the Chemical Sciences in Finding Alternatives to Critical Resources: A Workshop Summary; National Academies Press, 2012. 22916367

[ref12] Sample C. S.; Lee S.-H.; Li S.; Bates M. W.; Lensch V.; Versaw B. A.; Bates C. M.; Hawker C. J. Metal-Free Room-Temperature Vulcanization of Silicones via Borane Hydrosilylation. Macromolecules 2019, 52 (19), 7244–7250. 10.1021/acs.macromol.9b01585.

[ref13] Goswami K.; Skov A. L.; Daugaard A. E. UV-cured, platinum-free, soft poly(dimethylsiloxane) networks. Chemistry (Weinheim an der Bergstrasse, Germany) 2014, 20 (30), 9230–9233. 10.1002/chem.201402871. 25042056

[ref14] Li S.; Zhang J.; He J.; Liu W.; Wang Y.; Huang Z.; Pang H.; Chen Y. Functional PDMS Elastomers: Bulk Composites, Surface Engineering, and Precision Fabrication. Advanced Science 2023, 10 (34), e2304506 10.1002/advs.202304506. 37814364 PMC10700310

[ref15] Deriabin K. V.; Dobrynin M. V.; Islamova R. M. A metal-free radical technique for cross-linking of polymethylhydrosiloxane or polymethylvinylsiloxane using AIBN. Dalton Trans. 2020, 49 (26), 8855–8858. 10.1039/D0DT01061H. 32589173

[ref16] Mani S.; Cassagnau P.; Bousmina M.; Chaumont P. Cross-Linking Control of PDMS Rubber at High Temperatures Using TEMPO Nitroxide. Macromolecules 2009, 42 (21), 8460–8467. 10.1021/ma901521v.

[ref17] Zhao T.; Yu R.; Li S.; Li X.; Zhang Y.; Yang X.; Zhao X.; Wang C.; Liu Z.; Dou R.; Huang W. Superstretchable and Processable Silicone Elastomers by Digital Light Processing 3D Printing. ACS Appl. Mater. Interfaces 2019, 11 (15), 14391–14398. 10.1021/acsami.9b03156. 30912634

[ref18] Zheng S.; Zlatin M.; Selvaganapathy P. R.; Brook M. A. Multiple modulus silicone elastomers using 3D extrusion printing of low viscosity inks. Additive Manufacturing 2018, 24, 86–92. 10.1016/j.addma.2018.09.011.

[ref19] Wallin T. J.; Pikul J. H.; Bodkhe S.; Peele B. N.; Mac Murray B. C.; Therriault D.; McEnerney B. W.; Dillon R. P.; Giannelis E. P.; Shepherd R. F. Click chemistry stereolithography for soft robots that self-heal. J. Mater. Chem. B 2017, 5 (31), 6249–6255. 10.1039/C7TB01605K. 32264440

[ref20] Lu G.; Schneider A. F.; Vanderpol M.; Lu E. K.; Wong M. Y.; Brook M. A. Tunable, Catalyst-Free Preparation of Silicone Gels. Ind. Eng. Chem. Res. 2021, 60 (42), 15019–15026. 10.1021/acs.iecr.1c02369.

[ref21] Wong M. Y.; Schneider A. F.; Lu G.; Chen Y.; Brook M. A. Autoxidation: catalyst-free route to silicone rubbers by crosslinking Si–H functional groups. Green Chem. 2019, 21 (23), 6483–6490. 10.1039/C9GC03026C.

[ref22] So̷nderbæk-Jo̷rgensen R.; Meier S.; Dam-Johansen K.; Skov A. L.; Daugaard A. E. Reactivity of Polysilazanes Allows Catalyst-Free Curing of Silicones. Macromol. Mater. Eng. 2022, 307 (9), 2200157 10.1002/mame.202200157.

[ref23] Bui R.; Brook M. A. Dynamic covalent Schiff-base silicone polymers and elastomers. Polymer 2019, 160, 282–290. 10.1016/j.polymer.2018.11.043.

[ref24] Genest A.; Portinha D.; Fleury E.; Ganachaud F. The aza-Michael reaction as an alternative strategy to generate advanced silicon-based (macro)molecules and materials. Prog. Polym. Sci. 2017, 72, 61–110. 10.1016/j.progpolymsci.2017.02.002.

[ref25] Feng L.; Zhou L.; Feng S. Preparation and characterization of silicone rubber cured via catalyst-free aza-Michael reaction. RSC Adv. 2016, 6 (113), 111648–111656. 10.1039/C6RA23016D.

[ref26] Rambarran T.; Gonzaga F.; Brook M. A. Generic, Metal-Free Cross-Linking and Modification of Silicone Elastomers Using Click Ligation. Macromolecules 2012, 45 (5), 2276–2285. 10.1021/ma202785x.

[ref27] Gonzaga F.; Yu G.; Brook M. A. Polysiloxane Elastomers via Room Temperature, Metal-Free Click Chemistry. Macromolecules 2009, 42 (23), 9220–9224. 10.1021/ma902026j.

[ref28] Kroneková Z.; Mikulec M.; Petrenčíková N.; Paulovičová E.; Paulovičová L.; Jančinová V.; Nosál’ R.; Reddy P. S.; Shimoga G. D.; Chorvát D.; Kronek J. Ex Vivo and In Vitro Studies on the Cytotoxicity and Immunomodulative Properties of Poly(2-isopropenyl-2-oxazoline) as a New Type of Biomedical Polymer. Macromol. Biosci. 2016, 16 (8), 1200–1211. 10.1002/mabi.201600016. 27150385

[ref29] Paulovičová E.; Kroneková Z.; Paulovičová L.; Majerčíková M.; Kronek J. Cell-Mediated Immunoreactivity of Poly(2-isopropenyl-2-oxazoline) as Promising Formulation for Immunomodulation. Materials 2021, 14 (6), 1371 10.3390/ma14061371. 33809040 PMC7999147

[ref30] Jerca F. A.; Anghelache A. M.; Ghibu E.; Cecoltan S.; Stancu I.-C.; Trusca R.; Vasile E.; Teodorescu M.; Vuluga D. M.; Hoogenboom R.; Jerca V. V. Poly(2-isopropenyl-2-oxazoline) Hydrogels for Biomedical Applications. Chem. Mater. 2018, 30 (21), 7938–7949. 10.1021/acs.chemmater.8b03545.

[ref31] Zhang N.; Huber S.; Schulz A.; Luxenhofer R.; Jordan R. Cylindrical Molecular Brushes of Poly(2-oxazoline)s from 2-Isopropenyl-2-oxazoline. Macromolecules 2009, 42 (6), 2215–2221. 10.1021/ma802627y.

[ref32] Kronek J.; Minarčíková A.; Kroneková Z.; Majerčíková M.; Strasser P.; Teasdale I. Poly(2-isopropenyl-2-oxazoline) as a versatile functional polymer for biomedical applications. Polymers 2024, 16, 1708 10.3390/polym16121708. 38932057 PMC11207257

[ref33] Kopka B.; Kost B.; Basko M. Poly(2-isopropenyl-2-oxazoline) as a reactive polymer for materials development. Polym. Chem. 2022, 13 (33), 4736–4746. 10.1039/D2PY00660J.

[ref34] Fry E. M. Oxazoline Ring-Opening. J. Org. Chem. 1950, 15 (4), 802–806. 10.1021/jo01150a014.

[ref35] Spiridon M. C.; Jerca F. A.; Jerca V. V.; Vasilescu D. S.; Vuluga D. M. 2-Oxazoline based photo-responsive azo-polymers. Synthesis, characterization and isomerization kinetics. Eur. Polym. J. 2013, 49 (2), 452–463. 10.1016/j.eurpolymj.2012.11.024.

[ref36] Jerca V. V.; Nicolescu F. A.; Trusca R.; Vasile E.; Baran A.; Anghel D. F.; Vasilescu D. S.; Vuluga D. M. Oxazoline-functional polymer particles graft with azo-dye. React. Funct. Polym. 2011, 71 (4), 373–379. 10.1016/j.reactfunctpolym.2010.12.004.

[ref37] Merckx R.; Becelaere J.; Schoolaert E.; Frateur O.; Leiske M. N.; Peeters D.; Jerca F. A.; Jerca V. V.; de Clerck K.; Hoogenboom R. Poly(2-isoproprenyl-2-oxazoline)-Based Reactive Hydrophilic Cross-Linked Nanofiber Networks as the Basis for Colorimetric Continuous Meat Freshness Monitoring Sensors. Chem. Mater. 2023, 35 (17), 7079–7093. 10.1021/acs.chemmater.3c01355.

[ref38] Kopka B.; Kost B.; Pawlak A.; Tomaszewska A.; Krupa A.; Basko M. Covalent segmented polymer networks composed of poly(2-isopropenyl-2-oxazoline) and selected aliphatic polyesters: designing biocompatible amphiphilic materials containing degradable blocks. Soft Matter 2023, 19 (36), 6987–6999. 10.1039/D3SM00948C. 37667566

[ref39] Jerca F. A.; Jerca V. V.; Anghelache A. M.; Vuluga D. M.; Hoogenboom R. Poly(2-isopropenyl-2-oxazoline) as a versatile platform towards thermoresponsive copolymers. Polym. Chem. 2018, 9 (25), 3473–3478. 10.1039/C8PY00612A.

[ref40] Mazurek P.; Vudayagiri S.; Skov A. L. How to tailor flexible silicone elastomers with mechanical integrity: a tutorial review. Chem. Soc. Rev. 2019, 48 (6), 1448–1464. 10.1039/C8CS00963E. 30741275

[ref41] Raus V.; Hološ A.; Kronek J.; Mosnáček J. Well-Defined Linear and Grafted Poly(2-isopropenyl-2-oxazoline)s Prepared via Copper-Mediated Reversible-Deactivation Radical Polymerization Methods. Macromolecules 2020, 53 (6), 2077–2087. 10.1021/acs.macromol.9b02662.

[ref42] Kroneková Z.; Majerčíková M.; Paulovičová E.; Minarčíková A.; Danko M.; Markus J.; Letašiková S.; Kronek J. Cytotoxicity and bioimmunological activity of poly(2-isopropenyl-2-oxazoline) conjugates with ibuprofen using 3D reconstructed tissue models. Biomacromolecules 2024, 25, 3288–3301. 10.1021/acs.biomac.3c01434. 38805352

