# Supplementary material for: Metal-Free Curing of 3D Printable Silicone Elastomers via Thermally Triggered 2-Oxazoline Cross-Linkers
Source: Macromolecules. 2025 Feb 18;58(5):2709–18. doi: 10.1021/acs.macromol.4c03121 (PMC11912537; doi:10.1021/acs.macromol.4c03121)
Supplement: Supplementary file 1 — ma4c03121_si_001.pdf [file ma4c03121_si_001.pdf]

Supporting Information

Metal-free curing of 3D printable silicone elastomers via thermally-triggered 2-oxazoline cross-linkers

Paul Strasser[1], Christina Walliser[1], Edip Ajvazi[1], Felix Bauer[1], Oliver Brüggemann[1], Sebastian Lämmermann[2], Zoltan Major[2], Alžbeta Minarčíková[3], Monika Majerčíková[3], Matej Mičušík[3], Angela Kleinová[3], Zuzana Kroneková[3], Juraj Kronek[3] and Ian Teasdale[1]**

[1] Institute of Polymer Chemistry, Johannes Kepler University Linz, Altenberger Straße 69, 4040 Linz, Austria.

[2] Institute of Polymer Product Engineering, Johannes Kepler University Linz, Altenberger Straße 69, 4040 Linz, Austria.

[3] Department for Biomaterials Research, Polymer Institute, Slovak Academy of Sciences, 845 41 Bratislava, Slovakia.

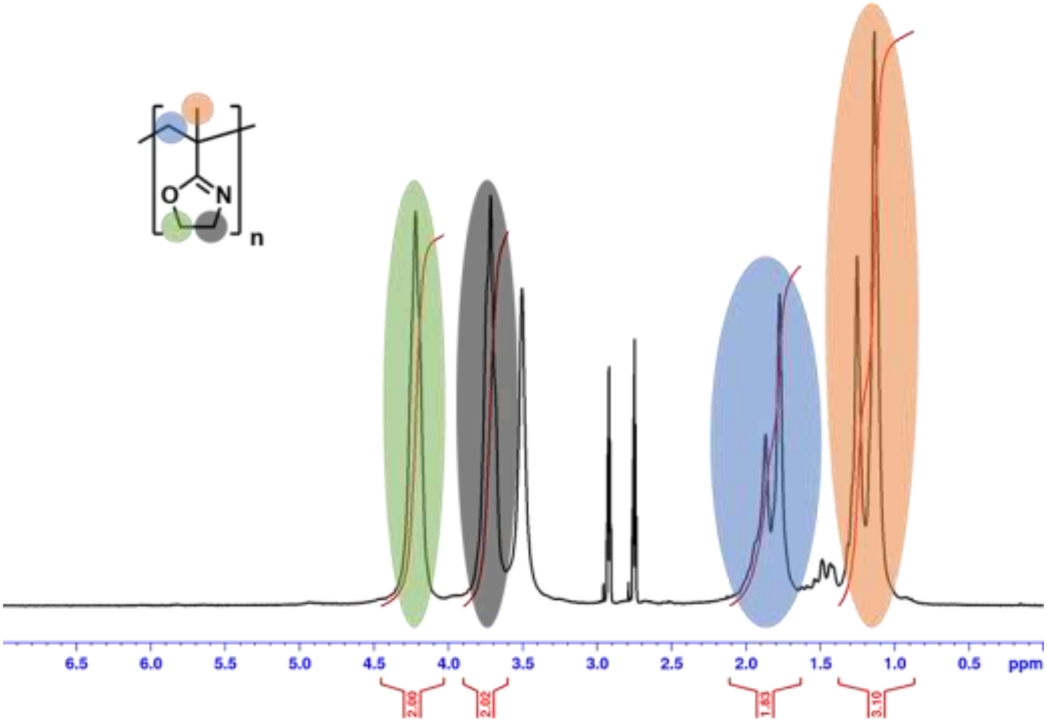

Figure S 1. ^{1}H-NMR spectrum in DMF-d_7 of PiPOx synthesized via a highly controlled copper (0)-mediated reversible-deactivation radical polymerization, showing water as impurity at around 3.5 ppm.

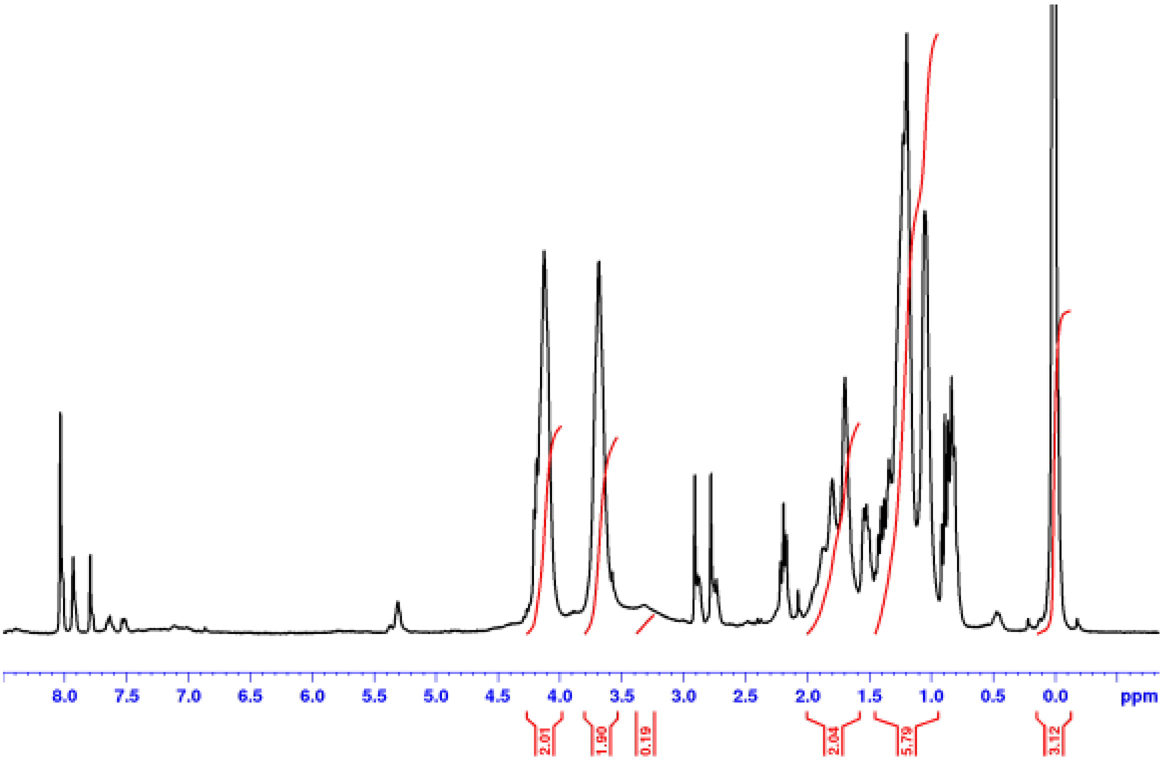

Figure S 2. ^{1}H-NMR spectrum in DMF-d_7 of the model reaction of PiPOx with PDMS-DMS-B12 at 5 % cross-linking density without any purification. The emerging peak at

around 3.3 ppm indicates successful ring opening of the pendant 2-oxazoline rings. Overlap with the neighboring signal, as well as the broadness leads to a distorted integral of the signal.

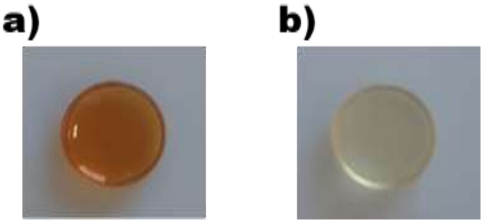

Figure S 3. Photographs of prepared elastomer pellets, a) E-B12/50% and b) E-B25/50%.

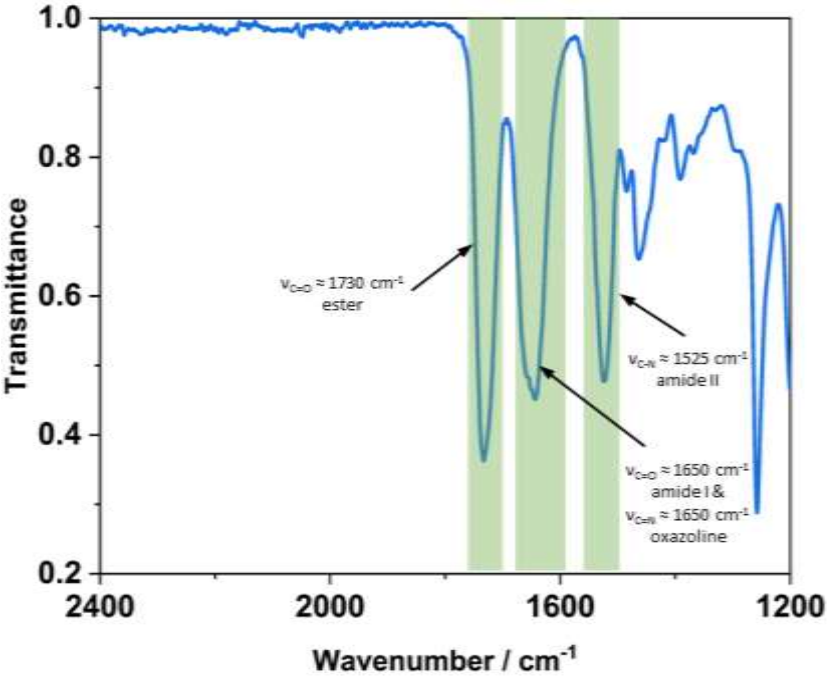

Figure S 4. FT-IR spectrum of elastomer E-B12/50% at 25 °C, showing the clear signal of the ester-amide linkage characteristic for the ring-opening reaction of the pendant 2-oxazoline rings of PiPOx.

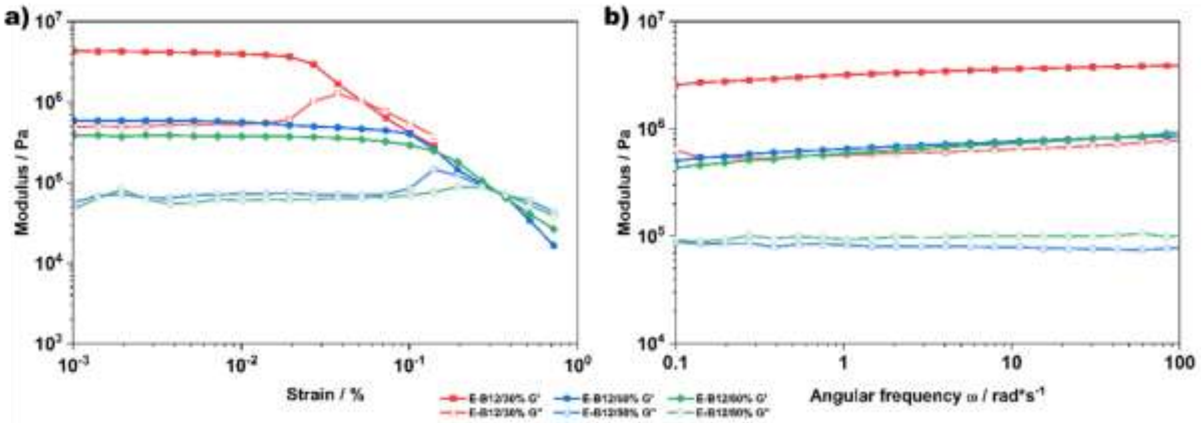

Figure S 5. Amplitude sweeps (a) of the elastomer series E-B12 with a constant frequency of 1 rad*s^{-1} and increasing strain rate from 10^{-3} % up to 10^{0} % or failure, as well as, the corresponding frequency sweeps (b).

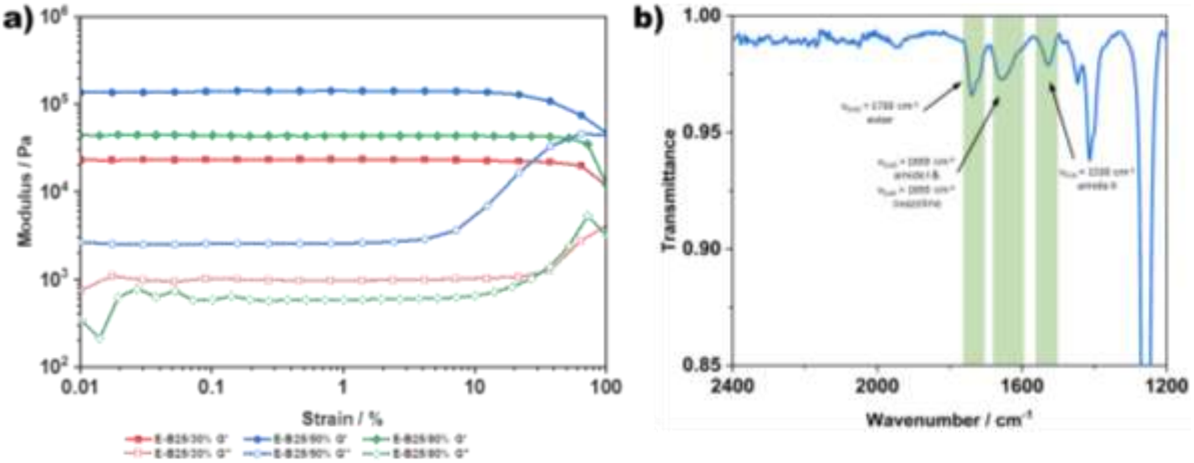

Figure S 6. a) Amplitude sweeps of the elastomer series E-B25 with a constant frequency of 1 rad*s^{-1} and increasing strain rate γ from $\gamma = 10^{-2}$ % up to 10^{2} %. b) FT-IR spectrum of elastomer E-B25/50% at 25 °C, showing the clear signal of the ester-amide linkage characteristic for the ring-opening reaction of the pendant 2-oxazoline rings of PiPOx.

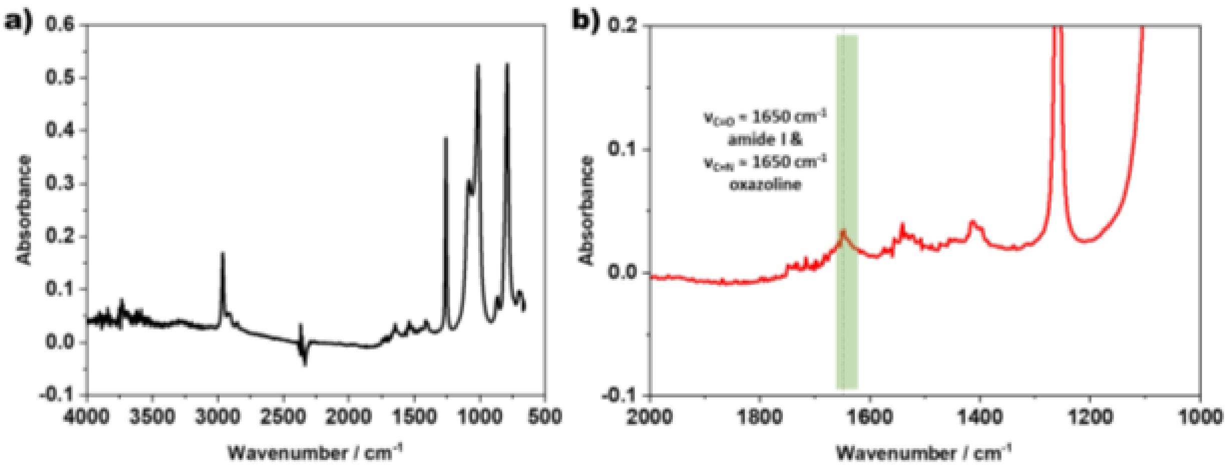

Figure S 7. a) Micro-ATR spectrum of E-B25/30% at 25 °C. c) Zoomed region of the micro-ATR-spectrum of E-B25/30%, showing the characteristic peak of the ester-amide-moiety formed upon ring opening of the 2-oxazoline ring of PiPOx.

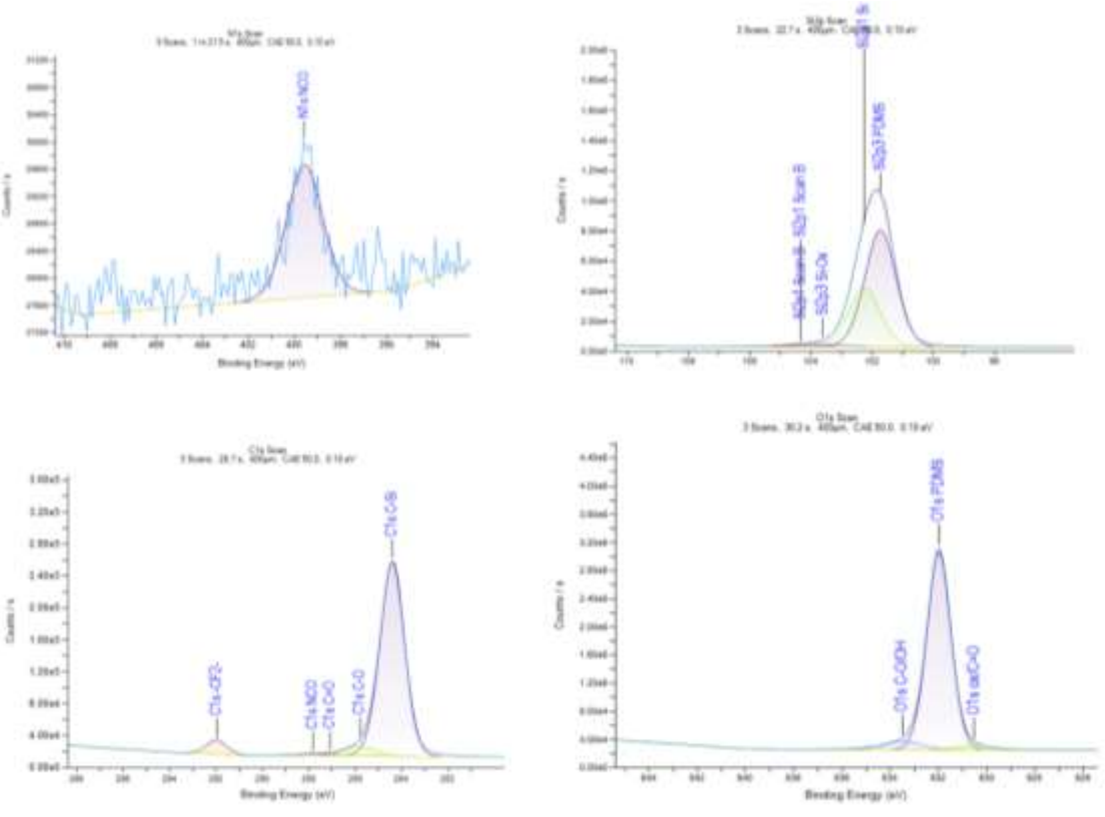

Figure S 8. Deconvolution of the XPS peaks for nitrogen, silicon, carbon and oxygen of E-B25/30%.

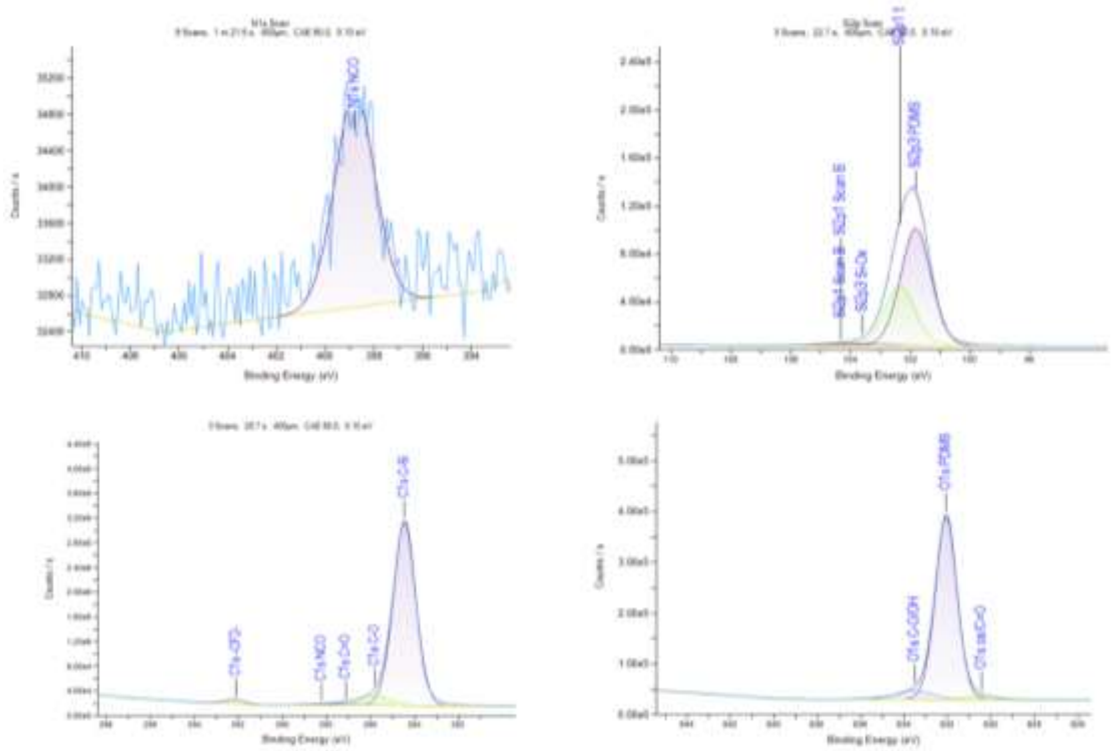

Figure S 9. Deconvolution of the XPS peaks for nitrogen, silicon, carbon and oxygen of E-B25/30% after washing in CH_2Cl_2.

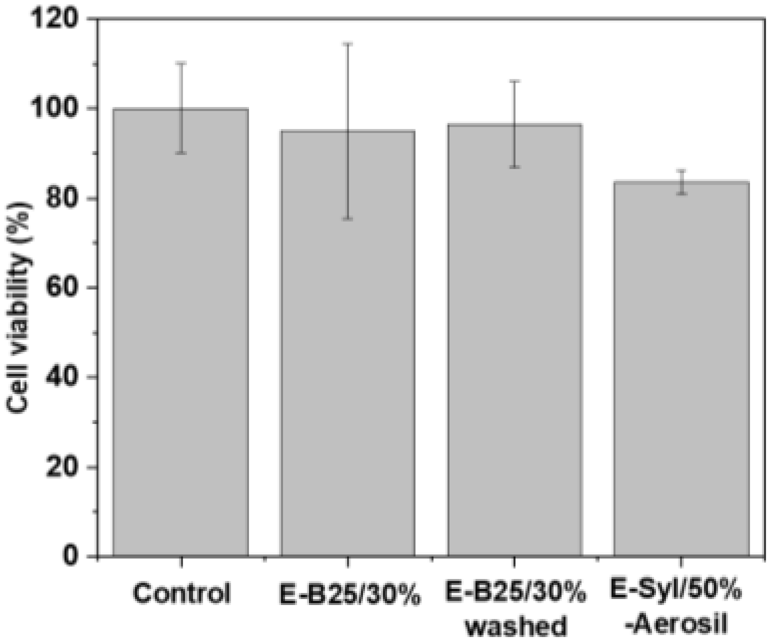

Figure S 10. In vitro contact cytotoxicity of elastomers E-B25/30%, before and after washing with CH_2Cl_2, and E-Syl/50%-Cytotox using mouse 3T3 fibroblasts.

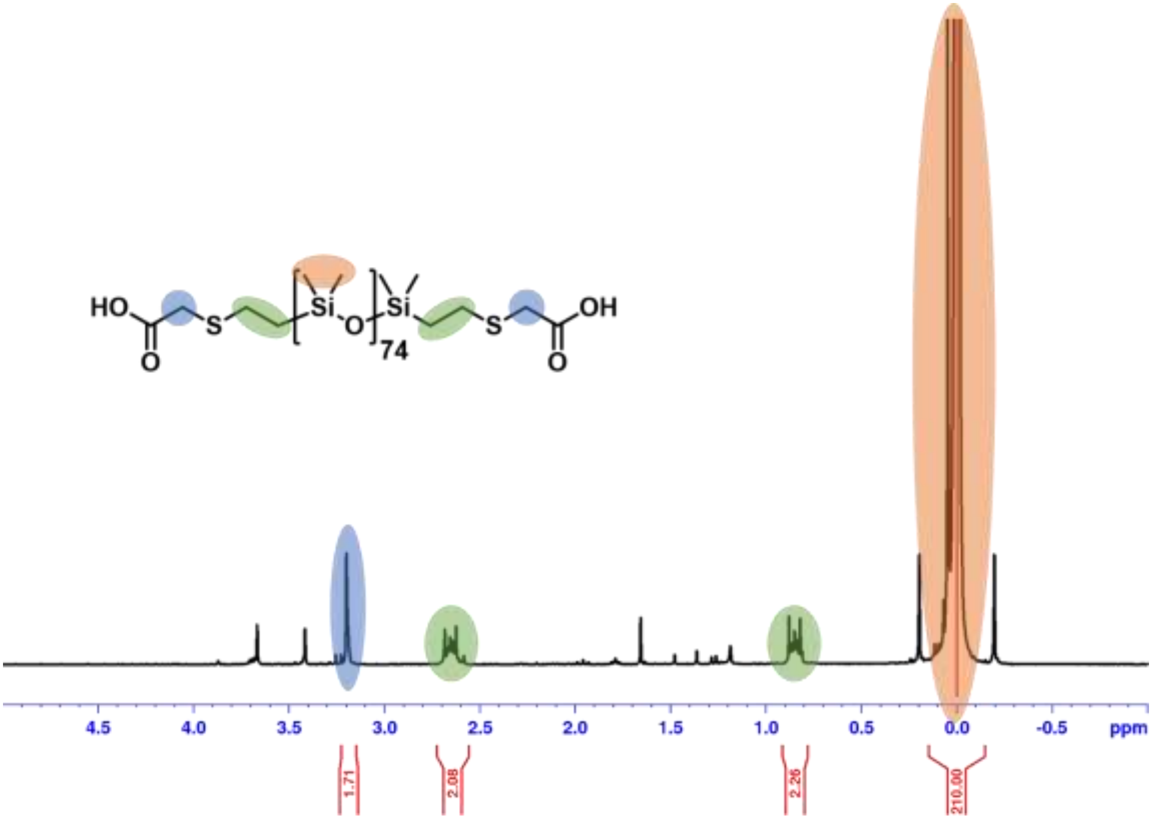

Figure S 11. ^{1}H-NMR spectrum of Syl-PDMS in $CDCl_3$, the thiol-ene reacted vinylterminated PDMS (100 cSt), showing the corresponding peaks of the newly functionalized end-groups.

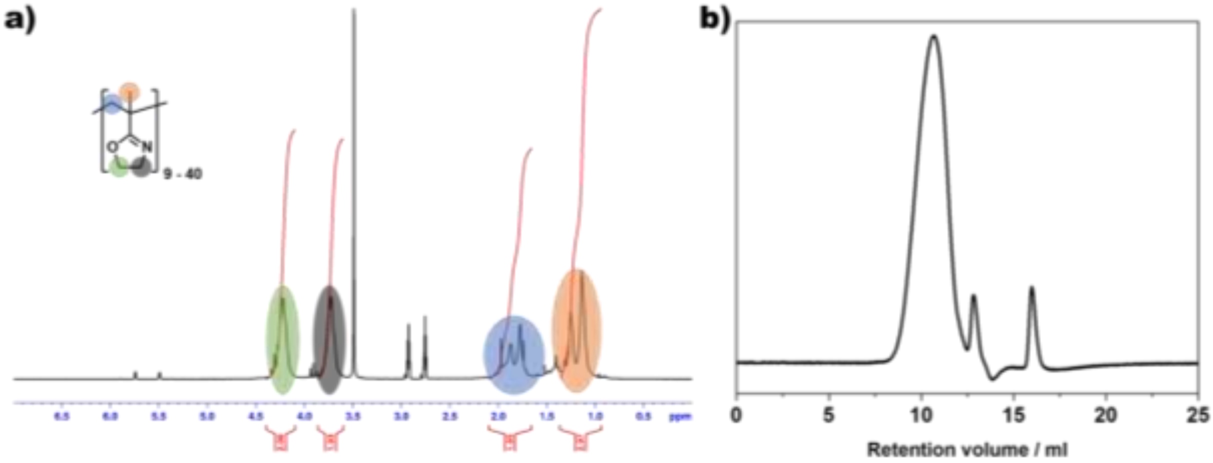

Figure S 12. ^{1}H-NMR spectrum in DMF-d_7 (a) and size exclusion chromatogram (b) of PiPOx synthesized via free-radical polymerization.

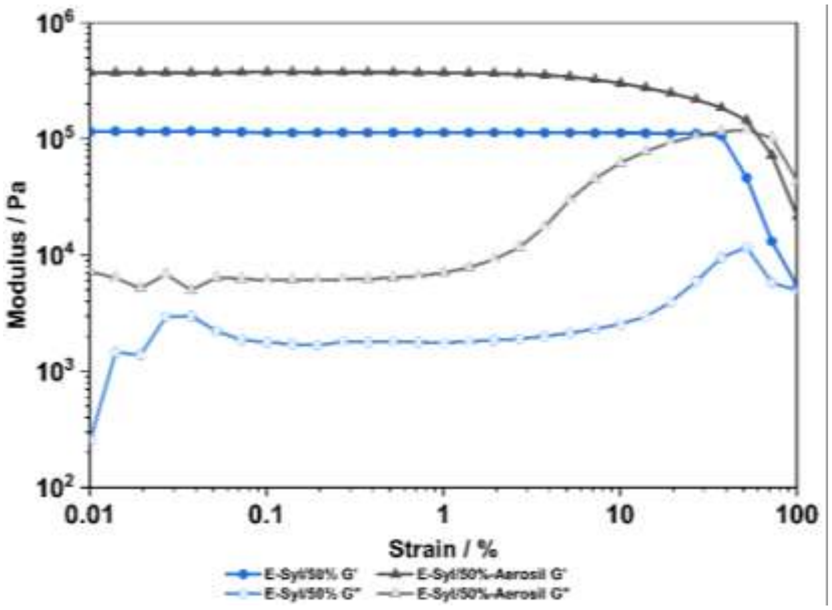

Figure S 13. Amplitude sweeps of the elastomer series E-B25 with a constant frequency of 1 rad*s^{-1} and increasing strain rate γ from $\gamma = 10^{-2}$ % up to 10^{2} %.

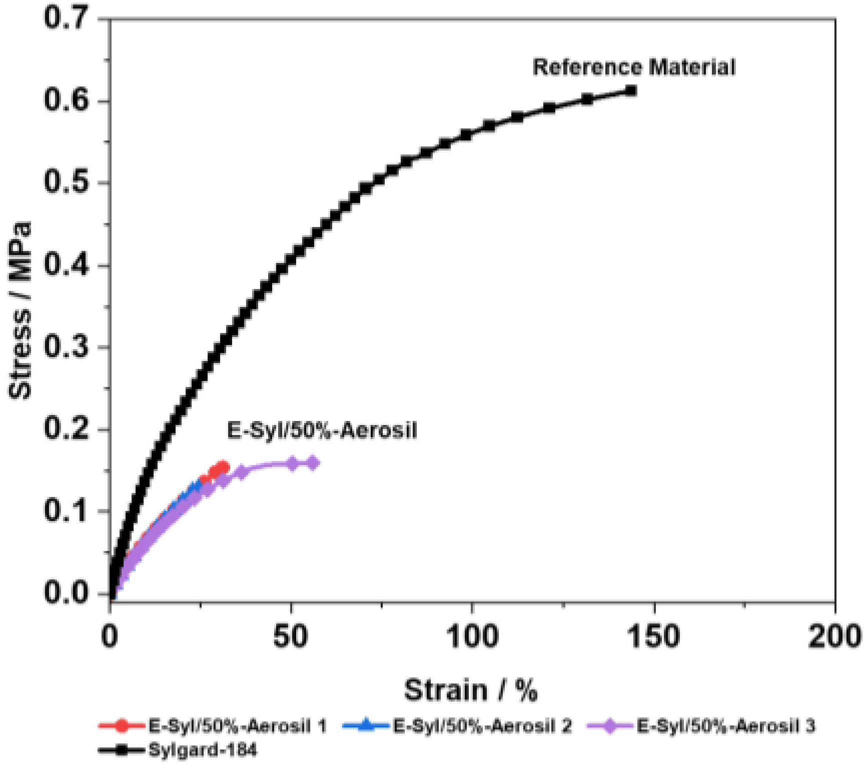

Figure S 14. Tensile tests (Dynamic mechanical analysis) of elastomer E-Syl/50%-Aerosil compared to Sylgard®-184 as reference. Sample E-Syl/50%-Aerosil 3 showed slight slipping at the end of the measurement.

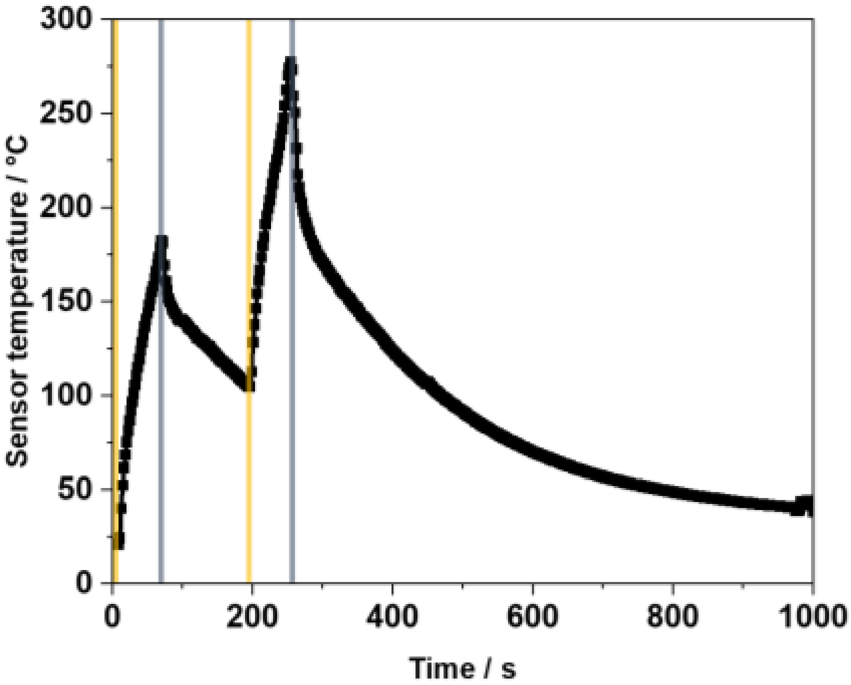

Figure S 15. Temperature profile during 3D-printing and curing via irradiation with a 2000 W halogen lamp, recorded 4 mm beneath the surface with a Type K thermocouple, connected to a Testo 176T4 temperature logger. Yellow bands indicate turning on the halogen lamp, dark blue bands indicate turning off the halogen lamp.

Table S 1. Apparent surface chemical composition of E-B25/30%, before and after washing in CH_2Cl_2, as determined by XPS.

	**C1s**	**Si2p**	**O1s**	**F1s**	**N1s**
	**C-Si/CO/C=O/NCO/CF$_2$**	**C-Si/Si-O**	**C=O/Si-O/C-O**	**F-/CF$_2$/CF$_3$**	**NC=O**
**E-B25/30%**	49.7	21.6	22.1	6.3	0.3
	43.7/2.6/0.1/0.6/2.7	20.7/0.9	0.8/19.9/1.4	0.4/5.6/0.3	
**E-B25/30% washed**	49.3	23.2	24.2	3.2	0.4
	44.2/3.0/0.5/0.6/1.0	22.3/0.9	0.7/21.7/1.8	0.1/2.8/0.3	

Table S 2. Gel fraction of the PiPOx-PDMS elastomers formulated based on the PDMS diacid PDMS-DMS-B12 and with increasing degree of 2-oxazoline ring-opening reaction.

Formulation	Number	Gel fraction / %	Average gel fraction / %
E-B12/30%	1	95	96
	2	100	
	3	94	
E-B12/50%	1	99	99
	2	99	
	3	99	
E-B12/80%	1	92	91
	2	92	
	3	90	

Table S 3. Gel fraction of the PiPOx-PDMS elastomers formulated based on PDMS-DMS-B25 and with increasing degree of 2-oxazoline ring-opening reaction.

Formulation	Number	Gel fraction / %	Average gel fraction / %
E-B25/30%	1	74	74
	2	73	
	3	75	
E-B25/50%	1	84	85
	2	86	
	3	86	
E-B25/80%	1	68	67
	2	67	
	3	67	

Table S 4. Gel fraction of the PiPOx-PDMS elastomers formulated based on functionalized Sylgard®184 Component A and 50 % of targeted 2-oxazoline ring-opening both without and with incorporated Aerosil® R 106 filler (10 wt%).

Formulation	Number	Gel fraction / %	Average gel fraction / %
E-Syl/50%	1	72	72
	2	72	
	3	71	
E-Syl/50%-Aerosil	1	88	88
	2	88	
	3	86	
